# Sleep-disordered breathing and metabolic syndrome across gender, age, and sleep subtypes in East Asians

**DOI:** 10.3389/fendo.2025.1519638

**Published:** 2025-07-14

**Authors:** Tong Feng, Qiong Ou, Guangliang Shan, Yaoda Hu, Huijing He

**Affiliations:** ^1^ Department of Respiratory and Critical Care Medicine, Deyang People’s Hospital, Affiliated Hospital of Chengdu College of Medicine, Deyang, China; ^2^ Sleep Center, Department of Respiratory and Critical Care Medicine, Guangdong Provincial People’s Hospital (Guangdong Academy of Medical Sciences), Southern Medical University, Guangzhou, China; ^3^ Department of Epidemiology & Biostatistics, Institute of Basic Medical Sciences, Chinese Academy of Medical Sciences/School of Basic Medicine, Peking Union Medical College, Beijing, China

**Keywords:** sleep-disordered breathing, metabolic syndrome, hypoxia, cardiovascular risk, gender differences, sleep quality, sleep subtypes

## Abstract

**Background:**

Previous research has classified sleep-disordered breathing (SDB) into various symptom phenotypes. The cardiovascular risks associated with SDB differ by gender, age, and subtype, with uncertainty about the mediating role of metabolic syndrome in these risks. This study has three primary objectives: (1) to analyze the correlation between SDB and MetS risk across gender-age groups and symptom subtypes, (2) to identify the nocturnal hypoxia parameter most indicative of this relationship, and (3) to evaluate the link between sleep parameters (insomnia, daytime sleepiness, snoring, and sleep duration) and MetS. Combining these parameters into a sleep quality score may improve predictions of health outcomes.

**Methods:**

Participants underwent type IV sleep monitoring and completed structured questionnaires. MetS was defined according to the Chinese Guidelines for the Prevention and Treatment of Type 2 Diabetes (2020 Edition). We conducted a latent class analysis to categorize patients based on SDB symptoms and characteristics.

**Results:**

The severity of SDB was independently associated with an increased risk of metabolic syndrome, particularly in males under 60 and females aged 60 and above. A total of 1,483 SDB patients were categorized into four distinct clusters: Cluster 1 included the pure insomnia group with fewer daytime symptoms; Cluster 2 consisted of the minimally symptomatic group; Cluster 3 comprised the insomnia group with multiple daytime symptoms; and Cluster 4 encompassed the group with upper airway symptoms and sleepiness. Among the SDB subtypes, there was no significant difference in the prevalence of metabolic syndrome. However, the pure insomnia group had the highest prevalence of hypertension.

**Conclusion:**

These findings highlight the importance of considering gender, age differences, and sleep symptom subtypes when evaluating and managing metabolic syndrome. Tailored strategies, early identification, and consideration of different subtypes are necessary to optimize treatment.

## 1 Introduction

Sleep-disordered breathing (SDB) is characterized by the repetitive narrowing and collapse of the upper airway, resulting in frequent hypoventilation or apnea during sleep (1). SDB and metabolic syndrome (MetS) share common pathogenic pathways, including hypertension, insulin resistance, and lipid metabolism abnormalities (2). Previous studies have indicated a complex, bidirectional causal relationship between these two conditions (3). The burden of hypoxia and frequent sleep disruptions caused by SDB may contribute to the onset of MetS, while the inflammatory responses and metabolic dysregulation associated with MetS could exacerbate SDB. Consequently, SDB and MetS often coexist. International epidemiological studies have revealed that the prevalence of MetS among SDB patients ranges from 55.8% to 79% (4–6), significantly higher than in the general population. In addition to shared pathogenic factors, SDB and MetS independently pose risks for various cardiovascular and metabolic disorders, with their co-occurrence synergistically increasing health risks (7).

Research findings indicate that the risks associated with SDB can differ across patient subgroups. For instance, Cano-Pumarega et al. observed in cohort studies that SDB is linked to an elevated risk of stage II hypertension in men but not in women (8). Similarly, Gottlieb et al. noted an association between SDB and an increased risk of coronary heart disease specifically in men under the age of 70, but not in older men or women of any age (9). Variations have been observed in studies focusing on the risk of stroke, atrial fibrillation, heart failure, and overall mortality (10–12). Previous research has categorized SDB symptoms into various phenotypes, each presenting distinct characteristics such as ‘minimally symptomatic,’ ‘excessive daytime sleepiness,’ ‘disturbed sleep,’ ‘upper airway symptoms,’ and ‘sleepiness dominant (13, 14).’ Interestingly, while traditional measures like the apnea-hypopnea index (AHI) do not differ greatly among SDB patients within these clusters, the potential outcomes of SDB, such as cardiovascular prognosis and treatment response, display differences (15). The cardiovascular risks associated with SDB exhibit variations based on gender, age, and subtype, with uncertainty over whether metabolic syndrome mediation influences this variability.

Existing research on individuals with SDB who reside in communities has predominantly concentrated on individuals with moderate to severe SDB, primarily focusing on older to middle-aged men (15, 16). Notably, there is a gap in the literature regarding the prevalence and characteristics of various SDB subtypes in Chinese community populations. Despite China having the highest number of individuals with SDB worldwide, it remains uncertain if SDB cohorts from Chinese communities exhibit similar symptom patterns. In addition, the association between different SDB subtypes and metabolic syndrome has been minimally explored in the literature.

The severity of SDB, as quantified by the AHI, shows limited predictive ability for adverse outcomes associated with SDB (17). Intermittent hypoxemia has been identified as a critical factor contributing to cardiovascular risk in individuals with SDB (18). However, there remains a lack of consensus regarding which parameters of nocturnal hypoxemia serve as the most sensitive and reliable predictors of metabolic syndrome, particularly in community-dwelling SDB patients. Nocturnal hypoxemia indicators can be easily derived from Type IV sleep monitors or consumer-grade sleep monitoring devices, making them suitable for screening SDB within the general population and potentially offering predictive markers for metabolic risks.

The primary clinical indications of SDB include daytime drowsiness and loud snoring. Some patients may also experience disrupted sleep patterns, difficulty falling asleep, trouble focusing, morning headaches, and persistent fatigue, along with other unusual symptoms. Research has identified a strong connection between daytime drowsiness, snoring, and specific metabolic disorders such as obesity, insulin resistance, and hypertension (19, 20). Current investigations often isolate specific facets of sleep rather than considering them in a holistic context. Past literature suggests that combining multiple sleep factors into an overall sleep quality score can provide insight into how sleep behaviors influence the development of metabolic syndrome (21–23). Therefore, a comprehensive understanding of the link between metabolic syndrome and an overall sleep quality score is crucial.

The severity of SDB is independently associated with an increased risk of MetS, which poses significant cardiovascular and metabolic risks. However, the relationship between SDB and MetS is complex and may vary across different demographic and symptom subgroups. The key hypotheses of this study are as follows: First, we hypothesize that SDB in Chinese community populations presents distinct symptom clusters, with varying associations to MetS across gender and age groups. Second, we hypothesize that specific sleep parameters, particularly nocturnal hypoxia indicators, are more predictive of MetS risk than traditional measures such as the AHI. Third, we propose that combining various sleep parameters into an overall sleep quality score will offer a more accurate prediction of MetS risk than evaluating each parameter individually. These hypotheses aim to provide new insights into the complex interactions between SDB, sleep quality, and metabolic health in diverse populations.

## 2 Materials and methods

### 2.1 Patients and study design

This study, a comprehensive, population-based survey, was conducted in Guangdong Province, Southern China, from April 9 to May 18, 2021. Known as the “Guangdong Sleep Health Study,” it was structured within the framework of the China National Health Survey (24). This study utilized a multi-stage, stratified, cluster sampling method to recruit a representative cohort based on demographic characteristics. Specifically, a three-level composite sampling framework (multi-stage, stratified, and cluster sampling) was implemented to ensure sample representativeness. Initially, we selected Shantou and Meizhou cities in Guangdong Province as the primary study areas. The research region was further divided into coastal (including Chenghai District and Jinping District in Shantou), island (including Nan’ao County in Shantou), and mountainous (including Meijiang District and Jiaoling County in Meizhou) areas, based on three distinct cultural backgrounds: Chaoshan, island, and Hakka.

Recruitment of participants began approximately one week before the formal survey. Participants were invited to schedule their participation via a WeChat mini-program module, which facilitated convenient and efficient appointment booking. With the assistance of local government authorities in the study areas, the research team invited residents from selected towns and communities to participate in the survey.

Initially, 5,838 participants completed the questionnaire survey. Of these, 3,829 completed sleep monitoring, while 2,009 did not. After excluding 133 participants with missing values for MetS, the number of remaining participants was reduced from 3,829 to 3,696. Further exclusions included 68 participants who had received OSA-related treatment, 102 who were using lipid-lowering drugs, and 87 who were using sedative hypnotic drugs. This resulted in 2,649 participants in the non- MetS group and 791 participants in the MetS group. The recruitment and enrollment process is detailed in **Supplementary Figure S1**.

The study received ethical approval from the Ethics Committee of Guangdong Provincial People’s Hospital (GDREC2020221H), ensuring adherence to ethical standards. Informed consent was obtained from all participants, guaranteeing their voluntary agreement to participate in the research.

### 2.2 Nighttime sleep study

In this study, a reliable type IV wearable sleep monitoring device (**Supplementary Figures S2, S3**), developed by Chengdu Cloud Care Healthcare Co. Ltd. in Chengdu, China, was used to monitor the sleep patterns of participants (25). This device has been previously described in research published by our group. Briefly, blood oxygen saturation is measured using the photoplethysmographic method, which relies on the absorption characteristics of hemoglobin for infrared and red light. The device records and analyzes effective monitoring time and the number of desaturation events (3%) using built-in automatic algorithms to generate a report. Monitoring is conducted on the thenar eminence of the palm, avoiding areas with veins, scars, spots, or dense hair.

This study compared and validated the Type IV sleep monitoring device against polysomnography at the Sleep Center of Guangdong Provincial People’s Hospital. A total of 196 participants were included, all of whom underwent an all-night PSG and simultaneous monitoring with the Type IV sleep monitoring device to assess its sensitivity and specificity in diagnosing OSA. When using an AHI of ≥5 as the diagnostic criterion for OSA, the sensitivity of the Type IV device was 93%, specificity was 77%, and the area under the curve (AUC) was 0.95. When the AHI threshold was raised to ≥15, the sensitivity was 92%, specificity was 89%, and the AUC remained 0.95 (25).

Among all individuals screened with the Type IV device, we selected 305 participants from Chenghai District and Jinping District in Shantou City and conducted home sleep apnea testing on them. The comparison of the consistency between the Type IV device and HSAT further validated the accuracy of the Type IV device in screening for OSA in a healthy community population. Among the 305 community participants validated with HSAT, the oxygen desaturation index (ODI) measured by the Type IV device showed a strong correlation with the AHI measured by HSAT (R²=0.504, P<0.001). Bland-Altman consistency testing indicated that approximately 93% (284/305) of the data points for the ODI from the Type IV device and the AHI from HSAT fell within the 95% limits of agreement (26).

Due to its ease of use, accessibility, and cost-effectiveness, the Type IV sleep monitoring device has been validated in various populations, including the general population, obese individuals, surgical patients, patients with acquired immune deficiency syndrome, and those with atrial fibrillation (27–30). The use of the Type IV sleep monitoring device alone or in combination with the Stop-Bang questionnaire showed significantly better performance in OSA screening compared to the Stop-Bang questionnaire alone, especially in detecting mild OSA (31). Despite some inconsistencies in the actual AHI estimates, the U.S. Preventive Services Task Force has stated that the Type IV sleep monitoring device generally demonstrates high accuracy in diagnosing OSA (32).

Various parameters such as 3% ODI, nocturnal mean oxygen saturation (MeanSpO2), lowest nocturnal oxygen saturation (MinSpO2), and proportion of time spent with oxygen saturation below 90% (T90) were recorded during the monitoring process. The presence and severity of SDB were determined based on ODI4% levels: normal (<5 events/h), mild (5 to <15 events/h), moderate (15 to <30 events/h), and severe (≥30 events/h) (28).

### 2.3 Definition of metabolic syndrome

According to the Chinese Guidelines for the Prevention and Treatment of Type 2 Diabetes (2020 Edition), metabolic syndrome is defined by the presence of at least three of the following components (33):

Hypertension: systolic blood pressure ≥130 mmHg, diastolic blood pressure ≥85 mmHg, or current use of antihypertensive medication;
Elevated triglyceride levels: ≥1.70 mmol/L;
Low high-density lipoprotein cholesterol levels: <1.04 mmol/L;
Abdominal obesity: waist circumference ≥85 cm for women or ≥90 cm for men;
Elevated fasting blood glucose levels: fasting blood glucose ≥6.1 mmol/L or current use of medication for hyperglycemia.

### 2.4 Physical examination

In this study, physical examinations included measurements of height, weight, neck circumference, waist circumference, blood pressure, and body composition analysis. All measurements were conducted by trained technicians and repeated twice to ensure accuracy.

Height was measured with participants standing next to a vertical wall on a level floor. During measurement, participants removed any hats and shoes, standing upright with the head, shoulders, buttocks, and heels touching the measuring instrument. The measuring plate was positioned parallel to the wall and precisely at the top of the participant’s head. Height was recorded in centimeters (cm), accurate to 0.1 cm.

Neck circumference and waist circumference were measured at the upper edge of the thyroid cartilage and 1 cm above the navel, respectively. Participants breathed naturally, avoiding deep inhalation, neck muscle tension, or abdominal contraction. A soft tape measure was placed close to the skin without compression, and measurements were recorded to the nearest 0.1 cm.

Weight was measured using the TANITA BC-420 body composition analyzer (manufactured in Japan). The instrument was placed on a flat surface and connected to a power source. Participants removed shoes and socks and stood with both feet evenly placed on the measurement plate. The researcher selected the appropriate measurement mode and input participants’ gender and height. The instrument completed the measurement within seconds, displaying parameters including weight, body fat percentage, muscle mass, and water content. Body Mass Index (BMI) was calculated by dividing weight by the square of height.

Blood pressure was measured three times using the Omron HEM-907 digital blood pressure monitor (manufactured in Japan), with each measurement taken 1 minute apart and the average value recorded. Before measurement, participants were advised to avoid stimulants such as caffeine and tobacco, intense exercise, and to rest quietly for 5 minutes. During measurement, participants sat comfortably with both feet flat on the floor and their left arm resting on a table or support. The cuff was positioned on the left arm at heart level, and the device automatically inflated to record real-time blood pressure data. After measurement, the device deflated and displayed the results, including systolic pressure, diastolic pressure, and heart rate.

### 2.5 Blood indicators

For each participant, sterile techniques were employed to extract 9 milliliters of fasting blood samples following an 8-hour fast, typically from the left anterior arm vein. Blood samples were collected in a specific sequence: first using the yellow blood collection tube, followed by the purple blood collection tube, which contained anticoagulants to prevent coagulation. After separation, the samples were stored at -20°C to ensure stability. During transportation, ice bags and sealed containers marked with biological hazard indicators were used to maintain safety and integrity from the collection point to the laboratory.

The analysis of the blood samples was conducted based on required indicators and methods. Conventional blood analysis was performed using the XT-4000i automated instrument, while specific biochemical analyses required special reagents and equipment. The routine blood analysis included measurements of red blood cells, white blood cells, platelets, and hemoglobin. Blood lipids were analyzed, including total cholesterol (TC), triglycerides (TG), low-density lipoprotein cholesterol (LDL-C), high-density lipoprotein cholesterol (HDL-C), and lipoprotein(a). Other biochemical indicators measured were fasting blood glucose, high-sensitivity C-reactive protein (hsCRP), alanine aminotransferase (ALT), aspartate aminotransferase (AST), urea, creatinine, and uric acid.

### 2.6 Structured questionnaire

This study utilized a standard questionnaire consisting of four parts. Each part of the questionnaire required signatures and special annotations from respondents, ensuring that final verifiers could quickly confirm the completion of the survey. The first part collected sociodemographic information, including age, gender, education level, and marital status. The second part gathered self-reported professional diagnoses of diseases such as hypertension, coronary heart disease, diabetes, and dyslipidemia, along with the disease history of individuals and their family members. The third part documented participants’ lifestyle habits, including smoking history, drinking history, and physical exercise. The fourth part recorded on-site physical examination results.

Smoking: Smoking continuously for more than six months. Quitting smoking refers to not smoking for at least six months after previously smoking. Drinking: Regularly drinking at least twice a month, with consumption of at least one bottle of beer or two liang of Baijiu/wine. Abstinence refers to not drinking for at least half a year. Physical Exercise: Engagement in physical exercise in the past year, averaging more than 20 minutes per session. Frequency categories are 5–7 days per week, 3–4 days per week, 1–2 days per week, ≤ 3 days per month, or never exercising. Hypertension: Use of antihypertensive drugs and/or systolic or diastolic blood pressure ≥ 140/90 mmHg. Diabetes: Fasting blood glucose ≥ 7 mmol/L or use of diabetes medications (oral hypoglycemic drugs and/or insulin).

The questionnaire was administered by a professional physician through face-to-face interviews. On-site supervisors conducted quality control inspections to ensure the completeness and authenticity of each questionnaire. At the end of each day, the questionnaires were scanned and converted into electronic format using specialized software, with data input checked for accuracy.

Participants with snoring and other sleep problems were evaluated using specific sleep questionnaires:

Epworth Sleepiness Scale (ESS): Developed by Murray W. Johns in 1991, this tool assesses daytime drowsiness tendencies with 8 descriptive questions, each scored from 0 to 3. Total scores range from 0 to 24, with a score of ≥ 9 indicating daytime sleepiness (34).

Pittsburgh Sleep Quality Index (PSQI): Compiled by sleep medicine experts from the University of Pittsburgh, the PSQI includes 19 items covering various aspects of sleep quality. Scores range from 0 to 21, with a score of ≤ 5 indicating good sleep quality and > 5 indicating poor sleep quality (35).

Insomnia Severity Index (ISI): Developed by Morin and Barlow in 1993, this scale assesses insomnia severity over the past week with 7 items, each scored from 0 to 4. Scores ≤ 7 indicate no insomnia, while scores > 7 indicate insomnia (36).

To evaluate the association between sleep parameters and the prevalence of MetS, an overall sleep quality score was calculated by combining all four sleep parameters, ranging from 0 (best) to 8 (worst), as detailed in **Supplementary Table S1**. In this study, the overall sleep score was considered both as a continuous variable and a categorical variable: scores > 5 were considered the worst sleep quality, 3–5 indicated poor sleep quality, and < 3 indicated the best sleep quality (37).

### 2.7 Statistical analysis

Quantitative data are presented as median ± interquartile range, and categorical data are presented as numbers (percentages). The comparison of quantitative data was performed using the independent samples t-test, while categorical data were compared using the chi-square test.

To evaluate the association between nocturnal hypoxia parameters, sleep symptoms, sleep patterns, and metabolic syndrome, univariate and multivariate logistic regression models were used. Model 1 is the unadjusted model, excluding covariates. Model 2 is the minimally adjusted model, adjusting only for sociodemographic variables. Model 3 is the fully adjusted model, adjusting for all covariates. To ensure the accuracy of the data analysis process, a sensitivity study was conducted. Oxygen saturation parameters were divided into quartiles, and the P-value for trend analysis was calculated to confirm the findings regarding oxygen saturation parameters as continuous variables and to explore any potential nonlinear relationships.

Due to the limitations of logistic regression models in handling nonlinear relationships, we used restricted cubic splines (RCS) to address potential nonlinear relationships between oxygen saturation parameters and metabolic syndrome. Subgroup analysis was performed using stratified binary logistic regression models, and the likelihood ratio test was used to investigate the impact of modifying subgroup indicators. Mediation analysis was conducted to determine the extent to which inflammation indices mediated the effect of the ODI on metabolic syndrome. The mediation analysis employed three equations to examine the relationships between the ODI, the mediator (inflammation indices), and the dependent variable (MetS). The analyses were adjusted for age, sex, marital status, education level, economic status, dietary factors (meat, seafood, egg, dairy, bean, vegetable, fruit, pickles), smoking, drinking, tea consumption, activities, and exercise. The proportion of mediation was used to evaluate the degree of the mediation effect.

Latent class analysis was performed using the ‘poLCA’ package in R software to fit 2 to 10 latent cluster solutions. The optimal number of clusters was determined by the smallest Bayesian Information Criterion (BIC). Once the optimal number of classes was determined, all variables were tested to identify significant differences among the clusters.

Statistical analyses were performed using the R software package (version 4.1.2) and Empower Stats (X&Y Solutions, Inc., Boston, MA).

## 3 Results

### 3.1 Characteristics of study participants

In the total population of 3,440 individuals, 791 (22.99%) have MetS (**Table 1**). Among the total population, 1,025 (29.80%) are male, and 2,415 (70.20%) are female. Individuals with MetS are generally older, have higher BMI and waist circumference, and exhibit significant differences in gender distribution, marital status, education level, and income compared to those without MetS. Additionally, they show distinct differences in lifestyle habits such as smoking and drinking, as well as in biochemical measurements including TG, HDL-C, LDL-C, and FPG levels.

**Table 1:** The characteristics of the study participants.

Variable	MetS			P-value
	Total Population, N = 3,440^1^	No, N = 2,649^1^	Yes, N = 791^1^	
Medical History and Physical Examination				
Age (years)	55 (46, 63)	52 (44, 61)	60 (53, 67)	<0.001
Gender [cases (%)]				<0.001
Male	1,025 (29.80%)	718 (27.10%)	307 (38.81%)	
Female	2,415 (70.20%)	1,931 (72.90%)	484 (61.19%)	
Marital Status [cases (%)]				<0.001
Single	183 (5.32%)	171 (6.46%)	12 (1.52%)	
Married	3,005 (87.35%)	2,312 (87.28%)	693 (87.61%)	
Divorced	40 (1.16%)	31 (1.17%)	9 (1.14%)	
Widowed	212 (6.16%)	135 (5.10%)	77 (9.73%)	
Education Level [cases (%)]				<0.001
Junior High School or Below	1,652 (48.02%)	1,188 (44.85%)	464 (58.66%)	
High School	819 (23.81%)	710 (26.80%)	109 (13.78%)	
College or Above	969 (28.17%)	751 (28.35%)	218 (27.56%)	
Monthly Per Capita Income (CNY)	3,000 (2,000, 4,527)	3,000 (2,000, 5,000)	2,500 (1,700, 3,927)	<0.001
Height (cm)	158 (153, 164)	158 (153, 163)	159 (154, 166)	0.002
Weight (kg)	59 (52, 66)	57 (51, 63)	65 (59, 73)	<0.001
BMI (kg/m²)	23.4 (21.3, 25.5)	22.7 (20.8, 24.6)	25.7 (24.1, 27.5)	<0.001
Neck Circumference (cm)	33.6 (31.6, 36.0)	33.0 (31.2, 35.2)	35.9 (33.8, 38.1)	<0.001
Waist Circumference (cm)	82 (75, 89)	79 (73, 85)	90 (86, 96)	<0.001
Smoking [cases (%)]				<0.001
Non-smoker	2,847 (82.76%)	2,249 (84.90%)	598 (75.60%)	
Former smoker	133 (3.87%)	88 (3.32%)	45 (5.69%)	
Current smoker	460 (13.37%)	312 (11.78%)	148 (18.71%)	
Alcohol Consumption [cases (%)]				<0.001
Non-drinker	2,876 (83.60%)	2,255 (85.13%)	621 (78.51%)	
Former drinker	58 (1.69%)	40 (1.51%)	18 (2.28%)	
Current drinker	506 (14.71%)	354 (13.36%)	152 (19.22%)	
Sleep Monitoring				
Oxygen Desaturation Index (events/h)	4.1 (2.1, 8.0)	3.7 (2.0, 7.1)	5.9 (3.0, 11.3)	<0.001
Average Arterial Oxygen Saturation (%)	96.72 (95.77, 97.46)	96.90 (95.98, 97.59)	96.15 (95.14, 96.94)	<0.001
Minimum Arterial Oxygen Saturation (%)	88.0 (84.0, 91.0)	88.0 (84.0, 91.0)	86.0 (83.0, 89.0)	<0.001
T90 (%)	1 (0, 4)	1 (0, 3)	2 (0, 7)	<0.001
Biochemical Measurements				
TG (mmol/L)	5.57 (4.87, 6.30)	5.53 (4.84, 6.22)	5.77 (4.97, 6.55)	<0.001
HDL-C (mmol/L)	1.38 (1.14, 1.64)	1.46 (1.24, 1.72)	1.05 (0.92, 1.31)	<0.001
LDL-C (mmol/L)	3.21 (2.65, 3.81)	3.19 (2.66, 3.76)	3.33 (2.64, 3.98)	0.011
TG (mmol/L)	1.20 (0.90, 1.80)	1.10 (0.80, 1.50)	2.00 (1.70, 2.80)	<0.001
FPG (mmol/L)	5.54 (5.18, 6.03)	5.43 (5.11, 5.78)	6.31 (5.67, 7.59)	<0.001
Diet and Lifestyle				
Meat Consumption [cases (%)]				0.363
5–7 days per week	3,080 (89.53%)	2,382 (89.92%)	698 (88.24%)	
3–4 days per week	244 (7.09%)	185 (6.98%)	59 (7.46%)	
1–2 days per week	90 (2.62%)	62 (2.34%)	28 (3.54%)	
≤3 days per month	14 (0.41%)	10 (0.38%)	4 (0.51%)	
Never	12 (0.35%)	10 (0.38%)	2 (0.25%)	
Seafood Consumption [cases (%)]				0.041
5–7 days per week	1,503 (43.69%)	1,149 (43.37%)	354 (44.75%)	
3–4 days per week	555 (16.13%)	433 (16.35%)	122 (15.42%)	
1–2 days per week	627 (18.23%)	499 (18.84%)	128 (16.18%)	
≤3 days per month	580 (16.86%)	448 (16.91%)	132 (16.69%)	
Never	175 (5.09%)	120 (4.53%)	55 (6.95%)	
Egg Consumption [cases (%)]				0.117
5–7 days per week	1,805 (52.47%)	1,410 (53.23%)	395 (49.94%)	
3–4 days per week	834 (24.24%)	638 (24.08%)	196 (24.78%)	
1–2 days per week	618 (17.97%)	465 (17.55%)	153 (19.34%)	
≤3 days per month	150 (4.36%)	107 (4.04%)	43 (5.44%)	
Never	33 (0.96%)	29 (1.09%)	4 (0.51%)	
Dairy Products [cases (%)]				0.014
5–7 days per week	757 (22.01%)	596 (22.50%)	161 (20.35%)	
3–4 days per week	471 (13.69%)	368 (13.89%)	103 (13.02%)	
1–2 days per week	688 (20.00%)	549 (20.72%)	139 (17.57%)	
≤3 days per month	842 (24.48%)	641 (24.20%)	201 (25.41%)	
Never	682 (19.83%)	495 (18.69%)	187 (23.64%)	
Soy Products [cases (%)]				0.331
5–7 days per week	420 (12.21%)	321 (12.12%)	99 (12.52%)	
3–4 days per week	629 (18.28%)	483 (18.23%)	146 (18.46%)	
1–2 days per week	1,535 (44.62%)	1,205 (45.49%)	330 (41.72%)	
≤3 days per month	751 (21.83%)	563 (21.25%)	188 (23.77%)	
Never	105 (3.05%)	77 (2.91%)	28 (3.54%)	
Vegetables [cases (%)]				0.713
5–7 days per week	3,346 (97.27%)	2,578 (97.32%)	768 (97.09%)	
3–4 days per week	68 (1.98%)	50 (1.89%)	18 (2.28%)	
1–2 days per week	26 (0.76%)	21 (0.79%)	5 (0.63%)	
Fruit [cases (%)]				0.150
5–7 days per week	1,712 (49.77%)	1,321 (49.87%)	391 (49.43%)	
3–4 days per week	663 (19.27%)	525 (19.82%)	138 (17.45%)	
1–2 days per week	726 (21.10%)	559 (21.10%)	167 (21.11%)	
≤3 days per month	292 (8.49%)	211 (7.97%)	81 (10.24%)	
Never	47 (1.37%)	33 (1.25%)	14 (1.77%)	
Pickled Products [cases (%)]				<0.001
5–7 days per week	341 (9.91%)	231 (8.72%)	110 (13.91%)	
3–4 days per week	273 (7.94%)	209 (7.89%)	64 (8.09%)	
1–2 days per week	614 (17.85%)	480 (18.12%)	134 (16.94%)	
≤3 days per month	1,519 (44.16%)	1,172 (44.24%)	347 (43.87%)	
Never	693 (20.15%)	557 (21.03%)	136 (17.19%)	
Tea Drinking [cases (%)]				<0.001
Non-drinker	802 (23.31%)	653 (24.65%)	149 (18.84%)	
Former drinker	19 (0.55%)	17 (0.64%)	2 (0.25%)	
Current drinker	2,619 (76.13%)	1,979 (74.71%)	640 (80.91%)	
Physical Activity [cases (%)]				0.243
Light	3,080 (89.53%)	2,382 (89.92%)	698 (88.24%)	
Moderate	253 (7.35%)	184 (6.95%)	69 (8.72%)	
Heavy	107 (3.11%)	83 (3.13%)	24 (3.03%)	
Exercise [cases (%)]				<0.001
5–7 days per week	1,763 (51.25%)	1,312 (49.53%)	451 (57.02%)	
3–4 days per week	372 (10.81%)	295 (11.14%)	77 (9.73%)	
1–2 days per week	394 (11.45%)	325 (12.27%)	69 (8.72%)	
≤3 days per month	317 (9.22%)	260 (9.82%)	57 (7.21%)	
Never	594 (17.27%)	457 (17.25%)	137 (17.32%)	
Metabolic Syndrome Assessment				
Central Obesity				<0.001
No	2,302 (68.11%)	2,106 (80.94%)	196 (25.19%)	
Yes	1,078 (31.89%)	496 (19.06%)	582 (74.81%)	
Hyperglycemia				<0.001
No	2,613 (75.96%)	2,325 (87.77%)	288 (36.41%)	
Yes	827 (24.04%)	324 (12.23%)	503 (63.59%)	
Hypertension				<0.001
No	1,945 (56.74%)	1,826 (69.14%)	119 (15.12%)	
Yes	1,483 (43.26%)	815 (30.86%)	668 (84.88%)	
Hypertriglyceridemia				<0.001
No	2,421 (70.40%)	2,228 (84.14%)	193 (24.40%)	
Yes	1,018 (29.60%)	420 (15.86%)	598 (75.60%)	
Low HDL Cholesterolemia				<0.001
No	2,899 (84.27%)	2,486 (93.85%)	413 (52.21%)	
Yes	541 (15.73%)	163 (6.15%)	378 (47.79%)	

^1^Median (Interquartile Range); n (%).

**Figure 1:**
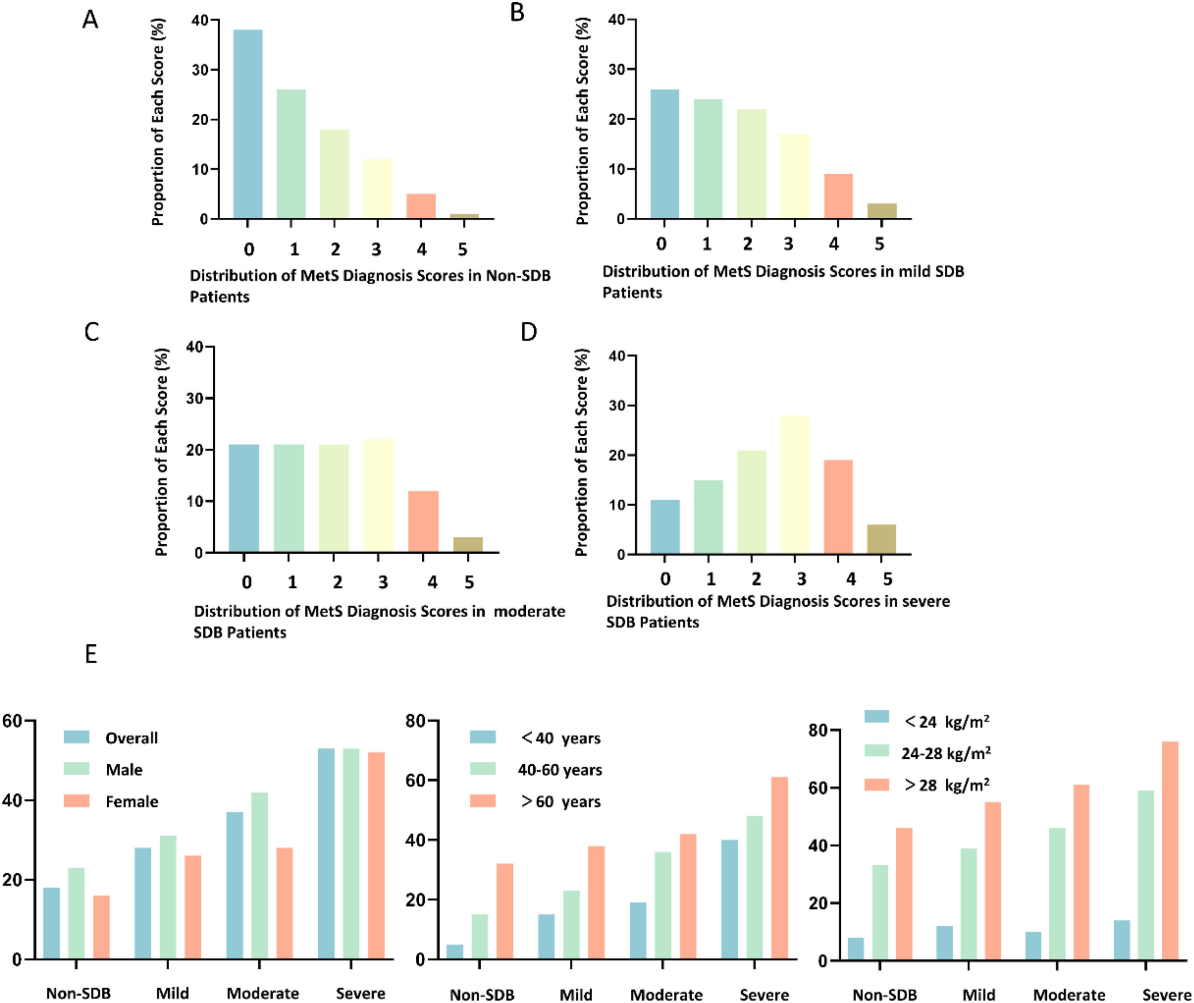
Distribution of Metabolic Syndrome (MetS) Scores in Subgroups with Different Severity of Sleep-Disordered Breathing (SDB). **(A)** Distribution of MetS Diagnosis Scores in Non-SDB Patients; **(B)** Distribution of MetS Diagnosis Scores in Mild SDB Patients; **(C)** Distribution of MetS Diagnosis Scores in Moderate SDB Patients; **(D)** Distribution of MetS Diagnosis Scores in Severe SDB Patients; **(E)** Changes in the Prevalence of MetS with the Severity of SDB in Subjects with Different Gender, Age, and Body Mass Index (BMI).

MetS scores show a significant increasing trend with the severity of SDB. The prevalence of MetS also increases significantly with the severity of SDB (**Figure 1**).

### 3.2 Association between SDB and MetS across different gender-age subgroups

The results show that the adjusted odds ratio (OR) for the risk of MetS in males with severe SDB is 4.50 [2.1, 9.6], and in females, it is 3.60 [1.4, 9.5] (**Table 2**). Further interaction analysis revealed that the difference in adjusted OR between gender subgroups was not statistically significant (p-value for gender difference = 0.302).

**Table 2:** Severity of SDB and risk of metabolic syndrome (Classified by Gender).

Severity of SDB	Adjusted OR (95% Confidence Interval)		p-value for Gender Difference
	Male	Female	
Non-SDB	1.0 (reference)	1.0 (reference)	0.3020
Mild	1.6 (1.1, 2.2) 0.006	1.5 (1.2, 1.9) <0.001	
Moderate	2.7 (1.8, 4.2) <0.001	1.7 (1.0, 2.8) 0.044	
Severe	4.5 (2.1, 9.6) <0.001	3.6 (1.4, 9.5) 0.010	
Trend p-value	<0.001	<0.001	

Age, sex, education level, marital status, smoking, alcohol consumption, tea drinking, diet, physical exercise, and physical labor were adjusted.

In males, the severity of SDB is independently associated with an increased risk of MetS in those under 60 years old (p-value for age group difference <0.001). In females, the severity of SDB is significantly associated with MetS in those aged 60 years and above (p-value for age group difference = 0.028) (**Table 3**). When classified by menopausal status, the severity of moderate to severe SDB in postmenopausal women is associated with metabolic syndrome (2.2 [1.3, 3.5], p = 0.002) (**Supplementary Table S2**).

**Table 3:** Risk of metabolic syndrome associated with the severity of SDB in different gender-age subgroups.

Adjusted OR (95% Confidence Interval)					P-value for Age Difference
Male	<40 years	40–49 years	50–59 years	≥60 years	
Severity of SDB					
Non-SDB	1.0 (reference)	1.0 (reference)	1.0 (reference)	1.0 (reference)	<0.001
Mild	190.0 (9.8, 3666.3) <0.001	8.1 (1.5, 42.8) 0.014	1.7 (0.8, 3.3) 0.140	1.0 (0.6, 1.7) 0.987	
Moderate to Severe	69.9 (3.5, 1402.7) 0.006	37.2 (5.8, 238.6) <0.001	5.3 (2.2, 12.3) <0.001	1.8 (1.0, 3.2) 0.066	
Trend p-value	0.003	<0.001	<0.001	0.140	
Female	<40 years	40–49 years	50–59 years	≥60 years	
Severity of SDB	1.0 (reference)	1.0 (reference)	1.0 (reference)	1.0 (reference)	0.028
Non-SDB	3.7 (0.6, 21.0) 0.144	2.7 (1.3, 5.6) 0.011	1.2 (0.8, 1.7) 0.406	1.6 (1.1, 2.3) 0.007	
Mild	0.0 (0.0, Inf) 0.998	1.4 (0.2, 9.3) 0.754	1.4 (0.6, 3.2) 0.408	2.8 (1.5, 5.3) 0.001	
Moderate to Severe	0.366	0.030	0.342	<0.001	

Age, sex, education level, marital status, smoking, alcohol consumption, tea drinking, diet, physical exercise, and physical labor were adjusted.

### 3.3 Association between SDB characteristic pathophysiological indicators and the prevalence of MetS

After adjusting for confounding factors such as age and gender, hypoxia burden indicators are associated with risk of MetS (**Table 4**). Using the low-risk group (Q1) as the reference, the risk of MetS for quartiles Q2 to Q4 of indicators reflecting hypoxia burden, such as the ODI, T90 was significantly increased (P<0.01) (**Supplementary Table S3**).

**Table 4:** Association between SDB characteristic pathophysiological indicators and the risk of metabolic syndrome.

Variables	Model1		Model2		Model3	
	OR (95%CI)	*P*	OR (95%CI)	*P*	OR (95%CI)	*P*
ODI	1.06 (1.05 - 1.07)	**<.001**	1.05 (1.04 - 1.06)	**<.001**	1.05 (1.04 - 1.06)	**<.001**
Meanspo2	0.76 (0.72 - 0.80)	**<.001**	0.84 (0.80 - 0.88)	**<.001**	0.84 (0.80 - 0.89)	**<.001**
Minspo2	0.94 (0.93 – 0.96)	**<.001**	0.96 (0.94 – 0.97)	**<.001**	0.96 (0.94 – 0.97)	**<.001**
T90	1.01 (1.01 – 1.01)	**<.001**	1.01 (1.00 – 1.01)	**<.001**	1.01 (1.00 – 1.01)	**<.001**

OR, Odds Ratio; CI, Confidence Interval.

Model 1: No covariates were adjusted.

Model 2: Only sociodemographic variables were adjusted (age, sex, education level, marital status).

Model 3: Model 2 variables plus smoking, alcohol consumption, tea drinking, diet, physical exercise, and physical labor were adjusted.Red values indicate P < 0.001.

**Figure 2:**
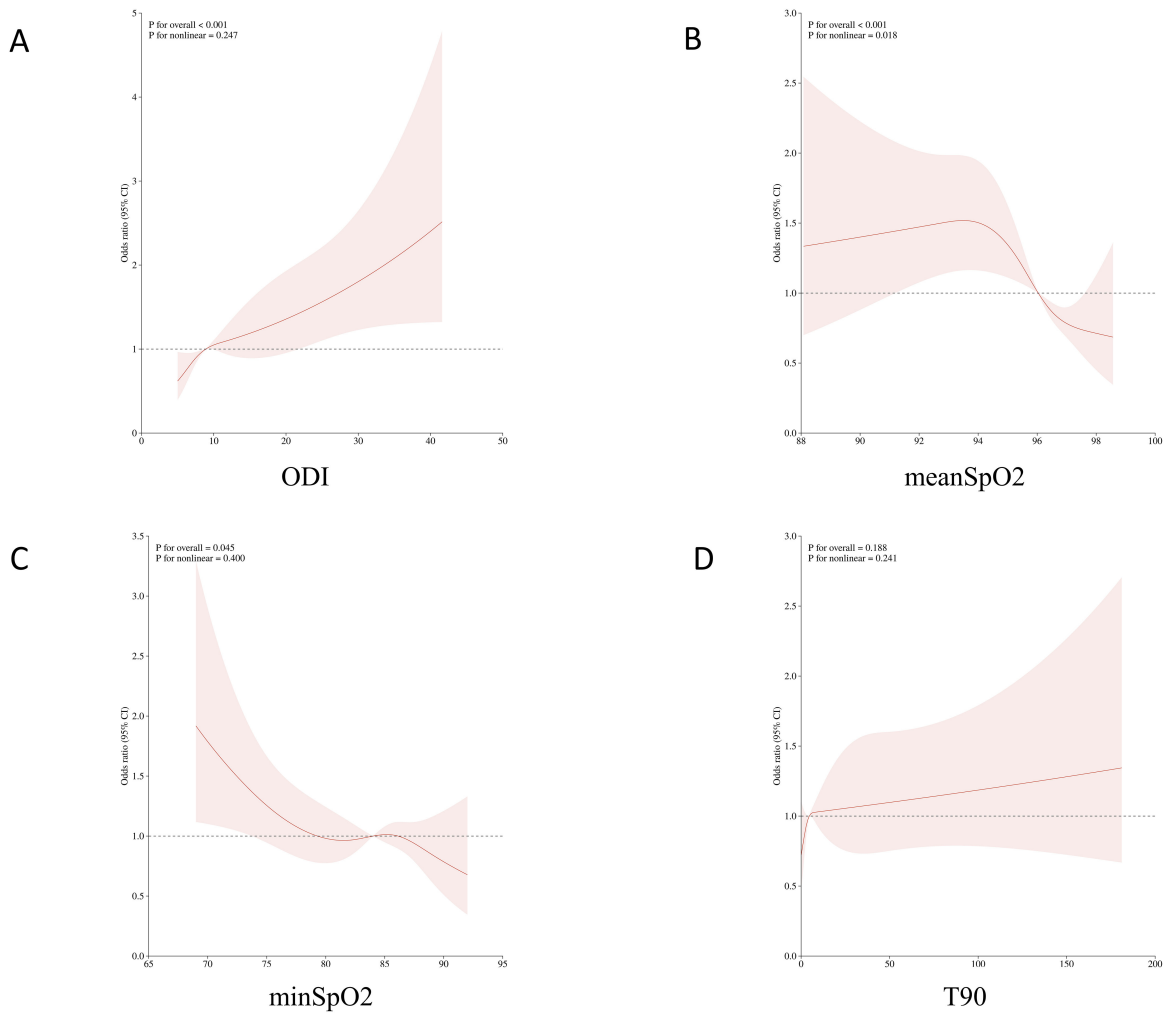
Non-linear associations between oxygen-related parameters and the risk of metabolic syndrome (MetS) using restricted cubic spline models. **(A)** Oxygen Desaturation Index (ODI); **(B)** Mean oxygen saturation (meanSpO?); **(C)** Minimum oxygen saturation (minSpO?); **(D)** Percentage of sleep time with oxygen saturation below 90% (T90).

In various subgroups, hypoxia burden indicators are associated with an increased prevalence of MetS in most subgroups (**Supplementary Tables S4, 5, 6, 7**). The nonlinear relationship between hypoxia burden indicators and prevalence of MetS was evaluated using RCS (**Figure 2**). The plot showed an inverted U-shaped association between continuous average arterial oxygen saturation and MetS. When MeanSpO2 was below 94%, the risk of MetS increased significantly.

**Figure 3:**
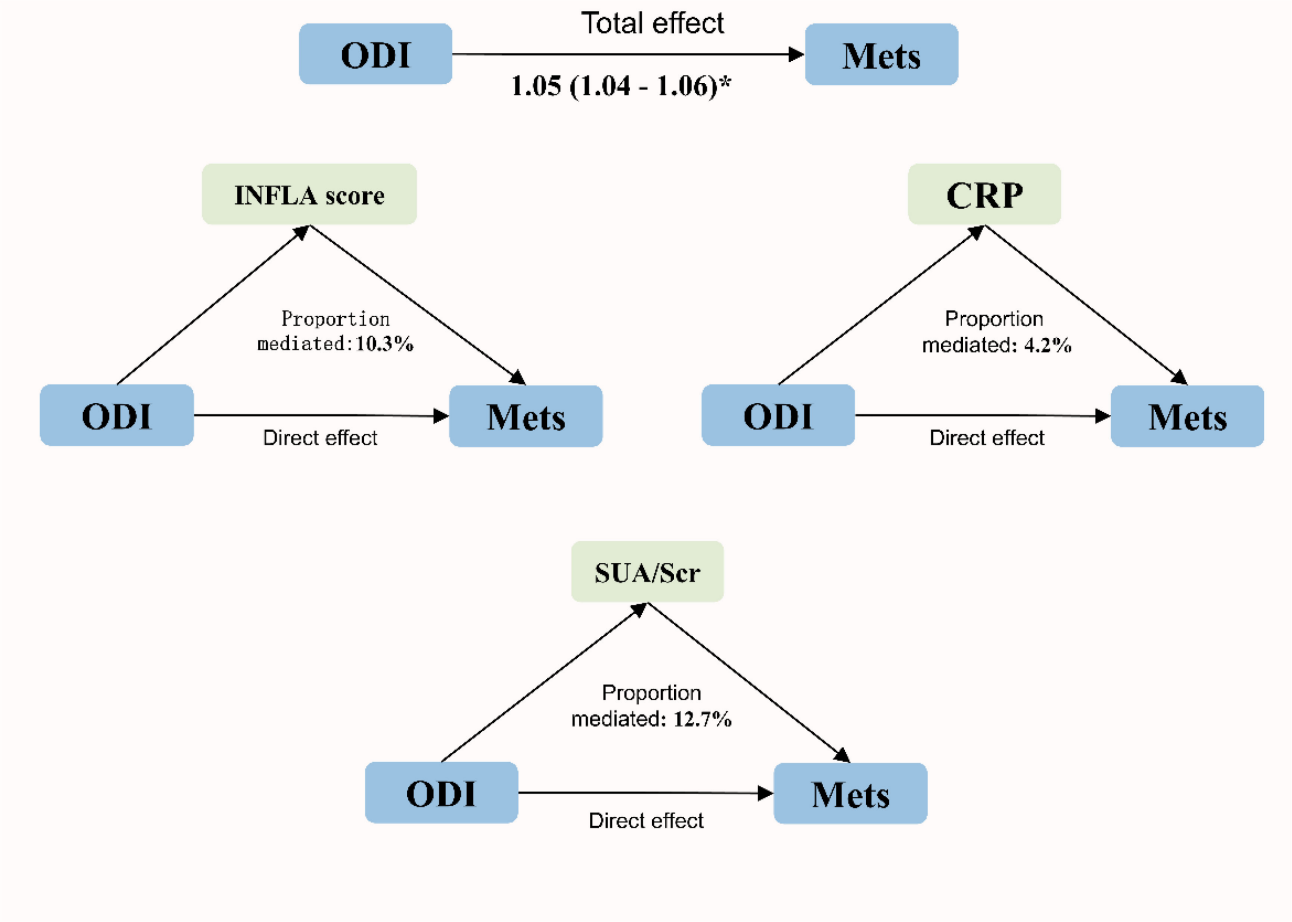
Mediation analysis for the associations between ODI and MetS.

The mediating effects of inflammation and uric acid on the association between ODI and MetS are shown in **Figure 3**. CRP, inflammation scores (INFLA), and uric acid played significant mediating roles in the association between ODI and MetS, with mediation ratios of 4.2%, 10.3%, and 12.7%, respectively (**Supplementary Table S8**).

### 3.4 The relationship between sleep symptoms and overall sleep quality with MetS

After controlling for covariates, regression analyses were conducted using the PSQI scale’s indicator of poor sleep quality, the overall scale score, seven component scores, sleep onset time, nighttime sleep duration, and nap duration as independent variables. Most of the associations between the variables and the research outcomes were not statistically significant, except for the use of hypnotic drugs (**Supplementary Table S9**). Similar results were observed for daytime sleepiness and insomnia when considered separately (**Supplementary Tables S10, S11**). Among symptoms related to SDB, loud snoring was associated with an OR for MetS of 2.02 (95% CI 1.57, 2.59), and observed apnea was associated with an OR for MetS of 1.62 (95% CI 1.09, 2.40) (**Supplementary Table S12**). Worse overall sleep quality was associated with a higher likelihood of having metabolic syndrome (OR 1.14, 95% CI 1.05, 1.23, p=0.0013 **Table 5**).

**Table 5:** Association between sleep quality and the risk of metabolic syndrome.

Variable	MetS	Abdominal Obesity	Hyperglycemia	Hypertension	High TG	Low HDL
Sleep score (continuous variable)	1.14 (1.05, 1.23) 0.0013	2.10 (1.81, 2.43) <0.0001	1.02 (0.93, 1.13) 0.6292	1.01 (0.94, 1.10) 0.7123	1.03 (0.94, 1.13) 0.5222	1.17 (1.03, 1.32) 0.0158
Sleep score (categorical variable)						
<3 (reference)	1.0	1.0	1.0	1.0	1.0	1.0
≥3, <5	1.23 (0.95, 1.60) 0.1158	3.06 (1.91, 4.90) <0.0001	0.84 (0.60, 1.17) 0.3045	0.82 (0.64, 1.07) 0.1431	0.91 (0.67, 1.24) 0.5448	1.34 (0.88, 2.05) 0.1716
≥5	1.77 (1.20, 2.62) 0.0041	12.95 (7.30, 22.97) <0.0001	1.19 (0.74, 1.92) 0.4740	1.20 (0.81, 1.77) 0.3681	1.27 (0.80, 2.00) 0.3130	1.72 (0.93, 3.18) 0.0821

Age, sex, education level, marital status, smoking, alcohol consumption, tea drinking, diet, physical exercise, and physical labor were adjusted.

**Figure 4:**
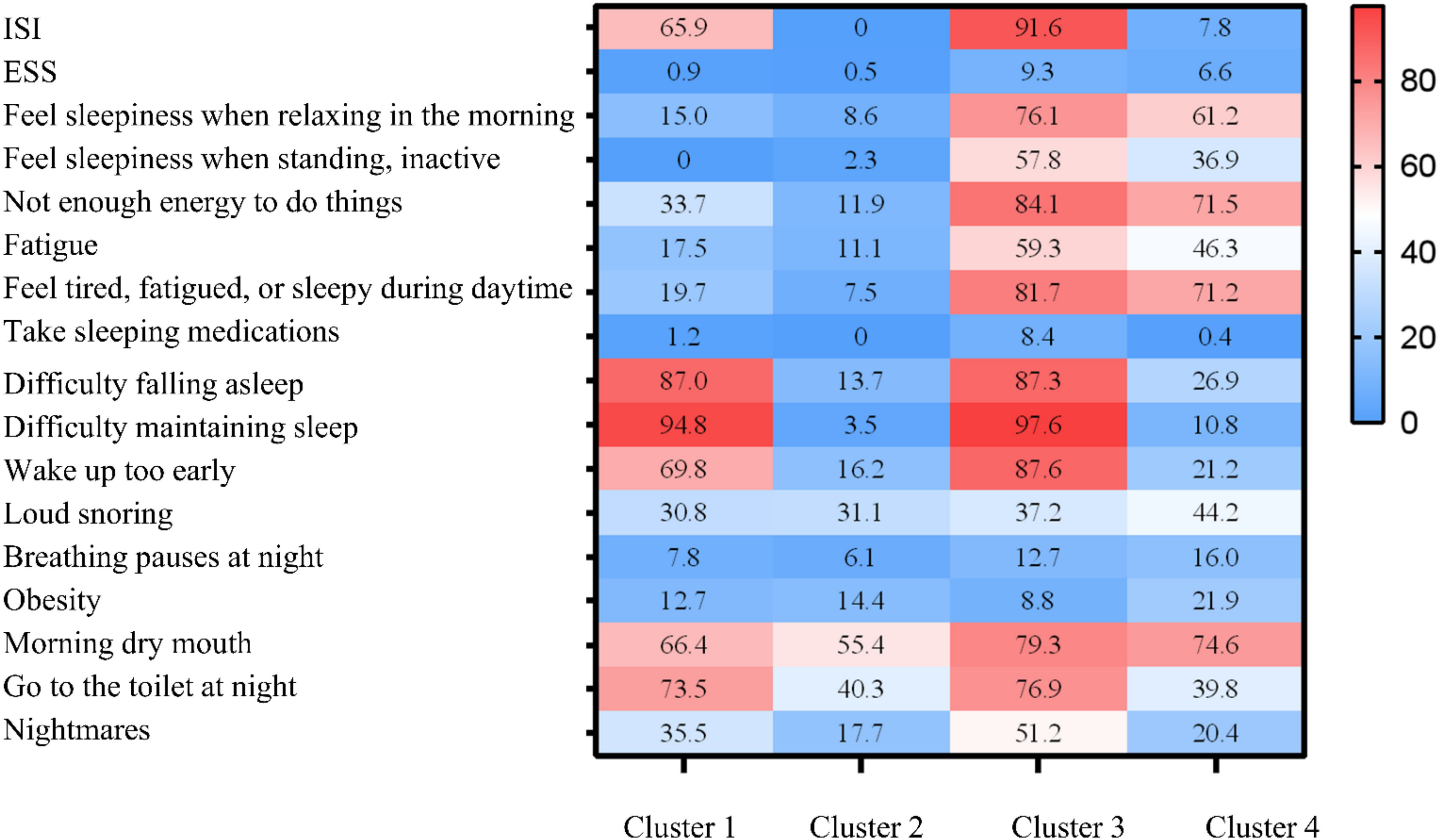
Profiles of the four sleep-disordered breathing (SDB) clusters. The relative differences in symptoms and signs among the four SDB clusters are shown in heatmaps. Cluster 1: Pure insomnia with fewer daytime symptoms group. Cluster 2: Minimally symptomatic group. Cluster 3: Insomnia with multiple daytime symptoms group. Cluster 4: Upper airway symptoms with sleepiness group. ESS, Epworth Sleepiness Scale; ISI, Insomnia Severity Index; SDB, sleep-disordered breathing.

Based on clinical interpretability, four subtypes were determined to be the most suitable clustering scheme. **Supplementary Table S13** presents the clinicodemographic participant characteristics in each cluster. We categorized the patients into four distinct clusters: Cluster 1 includes the pure insomnia group with fewer daytime symptoms; Cluster 2 consists of the minimally symptomatic group; Cluster 3 comprises the insomnia group with multiple daytime symptoms; and Cluster 4 encompasses the group with upper airway symptoms and sleepiness. **Figure 4** displays a heatmap of the frequency of specific symptoms and signs in each cluster, highlighting the major differences among the SDB subtypes. The proportion of comorbid hypertension was the highest in Cluster 1 (61.1%). There was no significant difference in the proportion of patients with comorbid MetS among the subtypes (**Table 6**).

**Table 6:** Metabolic syndrome and its components in each cluster.

Variable	Simple Insomnia	Mild Symptoms	Insomnia/More Daytime Symptoms	Upper Airway Symptoms with Sleepiness	P-value
Low HDL	50 (14.4%)	135 (21.6%)^a^	48 (19.1%)	64 (24.6%)^a^	0.010
Abdominal Obesity	158 (45.7%)	253 (41.9%)	92 (36.8%)	116 (44.8%)	0.145
Hyperglycemia	116 (33.4%)	162 (25.9%)	71 (28.3%)	52 (20.0%) ^a^	0.003
Hypertension	209 (61.1%)	320 (51.4%)^a^	107 (42.6%)^a^	114 (44.2%)^a^	<0.001
High TG	118 (34.0%)	216 (34.6%)	73 (29.1%)	84 (32.3%)	0.452
MetS	114 (32.9%)	193 (30.9%)	67 (26.7%)	74 (28.5%)	0.372

Compared with the simple insomnia group,

a P<0.05; compared with the mild symptoms group,

b P<0.05; compared with the insomnia/more daytime symptoms group,

c P<0.05.

## 4 Discussion

The prevalence of MetS among SDB patients reported in this study is 31.3%, which is lower than in some previous studies. Agrawal et al. conducted a survey in Indian and found that the prevalence of MetS among severe SDB patients was as high as 79% (5). Coughlin et al. in the UK reported an MetS prevalence of 87% among SDB patients (38). Wu et al. conducted a study in China and found that the prevalence of MetS among mild-moderate SDB patients was 52.4%, and as high as 58.7% among severe SDB patients (4). The differences in prevalence across studies may be related to the characteristics of the included populations. Previous studies often included high-risk groups for MetS, such as drivers and manual laborers. In contrast, this study consecutively included community-dwelling individuals, with a higher proportion of females, younger age, and lower BMI, resulting in a relatively lower prevalence of MetS compared to other studies.

In this large cross-sectional study, we explored the association between SDB and MetS based on demographic characteristics such as age and gender. The study found that in males, the severity of SDB is independently associated with an increased risk of MetS in those under 60 years old (p-value for age group difference <0.001). In females, the severity of SDB is significantly associated with MetS in those aged 60 years and above. The mechanisms underlying these gender differences are not yet clear. We hypothesize that they may be related to different patterns of sex hormone changes during the aging process in men and women (39). The age of 50 corresponds to the average menopausal age for women in South China, and menopause may simultaneously cause both SDB and MetS through mechanisms involving significant hormonal changes and inflammatory responses, thereby strengthening the relationship between the two conditions (40, 41). In men, there is a significant difference in the association between SDB and MetS among younger and older individuals. This could be due to the potentially milder impact of SDB-related damage in older patients. For instance, Kobayashi et al. found that, given the same severity of SDB, older patients experienced less intrathoracic negative pressure decline and lower arousal indices compared to younger patients (42). Consequently, the disease-related cardiac load and sympathetic-vagal imbalance might be less pronounced, making metabolic and cardiovascular damage less likely. Additionally, long-term exposure to SDB may result in “ischemic preconditioning,” potentially providing protective effects to organs. Regarding specific treatment responses and prognosis (43). Our findings suggest that CPAP (Continuous Positive Airway Pressure) intervention may yield more significant metabolic benefits in younger male patients and postmenopausal female patients.

We have identified four distinct subgroups of SDB within a general population residing in South China. Among these subtypes, we observed variations in the prevalence of hypertension comorbidities. Our findings align with existing literature, suggesting that the SDB subgroups primarily consist of individuals with minimal symptoms, indicating a lower overall symptom burden within the community of SDB patients. Notably, previous research, such as the Iceland Sleep Apnea Cohort study and the U.S. Sleep Heart Health Study (SHHS), categorized SDB patients into different subtypes based on clinical manifestations and comorbidities. For instance, the Iceland study identified three subtypes: “sleepy,” “disturbed sleep,” and “minimally symptomatic,” (13) while the SHHS study categorized patients into subtypes such as “insomnia,” “asymptomatic,” “moderately sleepy,” and “excessively sleepy (15).” China has the highest prevalence of sleep-disordered breathing (SDB) in the world, making it particularly important to study the characteristics of SDB in the Chinese population. Compared to other populations, Chinese SDB patients exhibit distinct differences in symptoms, clinical features, and associated comorbidities. Our study found that Chinese SDB patients show significantly different symptom patterns, particularly a higher proportion of “pure insomnia” and “minimally symptomatic” groups, where daytime symptoms are less prominent. This differs from findings in Western populations, where “daytime sleepiness” or “upper airway symptoms” are commonly seen in SDB patients. These differences may be closely related to the cultural background, lifestyle, and healthcare system in China.

For example, studies in Western countries typically find that daytime sleepiness is a prevalent symptom, and symptoms such as “sleep apnea” and “excessive daytime sleepiness” are considered key clinical features of SDB. However, in China, although SDB patients commonly experience nocturnal hypoxia and apnea, daytime sleepiness is not as significant a symptom. Instead, insomnia-related complaints are more prominent, suggesting that the manifestation of SDB may vary, which can lead to different diagnostic and treatment strategies. Furthermore, while there were no significant differences in nocturnal hypoxia parameters across the various SDB subtypes, we observed that Chinese patients, especially those in the “pure insomnia” group, had a higher prevalence of hypertension.

The HypnoLaus study with 1,377 participants and the Episono study with 476 participants, followed up for an average of (5.9 ± 1.3) years, found that moderate to severe SDB was associated with an increased risk of MetS (Metabolic Syndrome), with an OR and 95% confidence interval of 2.17 (1.41~3.36) (44). Among the indicators, T90 (cumulative time spent below 90% oxygen saturation) had the strongest association with the onset of MetS, with an OR and 95% confidence interval of 1.42 (1.04~1.95). This is consistent with the findings of Hirotsu et al. IH (Intermittent Hypoxia) is a fundamental characteristic of SDB and seems to be the main component triggering metabolic dysfunction. Both animal and clinical studies have shown that exposure to intermittent hypoxia can lead to sympathetic nervous system overactivity (45), systemic and vascular inflammation caused by NFkB (46), oxidative stress (47), and pro-inflammatory stimulation of adipose tissue through hypoxia-inducible factor 1 (HIF-1) (48). IH promotes hyperlipidemia in animal models by upregulating triglyceride and phospholipid biosynthesis pathways, inhibiting hepatic cholesterol uptake pathways, and reducing the clearance rate of triglyceride-rich lipoproteins (49). In terms of glucose metabolism, IH induces insulin resistance through β-cell dysfunction and adipose tissue inflammation (50). Compared to the control group, serum levels of hypoxia-inducible factor (HIF)-1α were significantly elevated in patients with type 2 diabetes (51). However, some previous interventional studies have shown that while CPAP treatment can significantly improve patient oxygenation, it does not significantly reduce individual components of MetS, has very weak effects on reducing visceral fat, and improves endothelial function (52). Although CPAP treatment is beneficial for metabolic abnormalities in patients with SDB, it may not be the most effective first-line treatment. Instead, medication and weight loss treatments might be more appropriate options. A 10-year follow-up study by Monneret on elderly SDB patients showed that the proportion of high-risk dyslipidemia patients was significantly lower in the statin-treated group compared to the untreated group (53). A comparison of the effects on metabolic variables within 24 weeks of weight loss alone or combined with CPAP treatment versus CPAP treatment alone found that CRP (C-reactive protein), insulin resistance, and triglyceride levels only decreased in the weight loss groups, while they remained unchanged in the group receiving CPAP treatment alone (54).

In a comprehensive community-based study involving more than 3,000 participants, it was discovered that individuals with higher scores indicating poor overall sleep quality were more likely to have MetS. Additionally, participants who frequently snored were found to have an increased likelihood of MetS. These findings are in line with previous research conducted by Troxel and colleagues, who investigated the link between sleep symptoms, such as insomnia and SDB, and MetS in a longitudinal study involving 812 American adults aged 45–75 years based on ATP III criteria. After adjusting for various factors, including demographics, lifestyle habits, and depressive symptoms, it was revealed that difficulties in falling asleep, non-restorative sleep, and loud snoring were significantly associated with a higher risk of developing MetS over a 3-year period. However, following further adjustments for AHI, only loud snoring emerged as a significant predictor of MetS incidence (OR 3.01, 95% CI 1.39-6.55), while difficulties in falling asleep and non-restorative sleep showed no significant associations (55). This finding suggests that the relationship between MetS and SDB may be more robust compared to that with insomnia symptoms.

Our research endeavors are bound by certain constraints. Firstly, this study adopts a cross-sectional design, which precludes the determination of cause-and-effect relationships for the identified associations. Secondly, the collection of all sleep-related data relies on self-reported information, rendering it susceptible to both recall bias and misclassification, particularly with regards to sleep duration. Additionally, the utilization of type IV wearable sleep monitors for diagnosing SDB may potentially underestimate the severity of the condition, especially among female participants. Consequently, the applicability of our findings may be constrained. Nonetheless, the extensive size of the study’s sample population likely mitigates the likelihood of such biases.

## 5 Conclusion

The coexistence of SDB and MetS significantly increases the health risks of cardiovascular and metabolic diseases, and comprehensive evaluation and management strategies need to consider differences in gender, age, and symptom subtypes. By integrating multiple sleep parameters into an overall sleep quality score, this method is more accurate in predicting health outcomes than evaluating each parameter individually. This overall evaluation helps to improve clinical practice and intervention strategies, ultimately improving patient health outcomes. Future research should further explore the mechanisms underlying gender and age-specific differences in the association between SDB and MetS. Longitudinal studies also need to establish causal relationships and evaluate the long-term impact of comprehensive sleep quality assessment on metabolic health.

## Data Availability

The raw data supporting the conclusions of this article will be made available by the authors, without undue reservation.
