# Supplementary material for: Sleep-disordered breathing and metabolic syndrome across gender, age, and sleep subtypes in East Asians
Source: Front Endocrinol (Lausanne). 2025 Jul 14;16:1519638. doi: 10.3389/fendo.2025.1519638 (PMC12301214; doi:10.3389/fendo.2025.1519638)
Supplement: Supplementary file 1 [file DataSheet1.docx]

Title “Sleep-disordered breathing and metabolic syndrome across gender, age, and sleep subtypes in East Asians: the Guangdong Sleep Health Study”

**Supplementary Materials**

**Supplementary Table Legend**

**Supplementary Table 1.**The overall sleep quality score scale according to each sleep parameter score

**Supplementary Table 2.** Risk of MetS Associated with the Severity of SDB (Classified by Menopausal Status)

**Supplementary Table** **3.** Association Between SDB Characteristic Pathophysiological Indicators (Quartiles) and the Risk of Metabolic Syndrome

**Supplementary Table 4.** Stratified analyses for the association between ODI and MetS.

**Supplementary Table 5.** Stratified analyses for the association between MeanspO2 and MetS.

**Supplementary Table 6.** Stratified analyses for the association between Minspo2 and MetS.

**Supplementary Table 7.** Stratified analyses for the association between T90 and MetS.

**Supplementary Table 8.** Mediation analysis for the associations between ODI and MetS.

**Supplementary Table 9.** Association Between Sleep Conditions and MetS Prevalence.

**Supplementary Table 10.** Association Between daytime sleepiness and MetS Prevalence.

**Supplementary Table 11.**Association Between insomnia and MetS Prevalence.

**Supplementary Table 12.**Association Between SDB-Related Symptoms and MetS Prevalence.

**Supplementary Table 13.**Clinicodemographic participant characteristics in each cluster

**Supplementary Figure Legend**

**Supplementary Figure 1.** Flowchart of participant selection

**Supplementary Figure 2.**Type IV intelligent wearable sleep monitoring devices and method of use

**Supplementary Figure 3.**Validation of intelligent wearable sleep monitoring devices by comparison against polysomnography

**Supplementary Table 1.**The overall sleep quality score scale according to each sleep parameter score

| Sleep parameters | Score | Overall sleep quality score |
| --- | --- | --- |
| Insomnia | ISI score  ≤ 7  > 7 | 0  2 |
| Daytime sleepiness | ESS score  <10  ≥10 | 0  2 |
| Sleep duration | 7-8hr/d  6-7hr/d  ≥8hr/d  <6hr/d | 0  1  2  2 |
| Snoring | No  Yes | 0  2 |
| Total score |  | 0 (best) -8 (worst) |

Footnotes: ISI: Insomnia Severity Index ; ESS: the Epworth Sleepiness Scale.

**Supplementary Table 2.** Risk of Metabolic Syndrome Associated with the Severity of SDB (Classified by Menopausal Status)

|  | Adjusted OR (95% Confidence Interval) | | |
| --- | --- | --- | --- |
|  | Male | Premenopausal Female | Postmenopausal Female |
| Severity of SDB |  |  |  |
| Non-SDB | 1.0 (reference) | 1.0 (reference) | 1.0 (reference) |
| Mild | 1.6 (1.1, 2.2) 0.006 | 2.1 (1.2, 3.7) 0.012 | 1.4 (1.1, 1.9) 0.006 |
| Moderate to Severe | 3.0 (2.0, 4.4) <0.001 | 1.1 (0.2, 5.7) 0.888 | 2.2 (1.3, 3.5) 0.002 |

**Supplementary Table** **3.** Association Between SDB Characteristic Pathophysiological Indicators (Quartiles) and the Risk of Metabolic Syndrome

|  | OR | 95%CI | P | OR(adjusted) | 95%CI(adjusted) | P (adjusted) |
| --- | --- | --- | --- | --- | --- | --- |
| ODI |  |  |  |  |  |  |
| Q1 | 1.0 (reference) |  |  | 1.0 (reference) |  |  |
| Q2 | 1.175 | [0.843,1.638] | 0.341 | 1.131 | [0.802,1.594] | 0.483 |
| Q3 | 1.664 | [1.201,2.305] | 0.002 | 1.542 | [1.100,2.162] | 0.012 |
| Q4 | 2.055 | [1.493,2.828] | 0.000 | 1.913 | [1.367,2.678] | 0.000 |
| P for trend |  |  | 0.000 |  |  | 0.000 |
| meanSpO2 |  |  |  |  |  |  |
| Q1 | 1.0 (reference) |  |  | 1.0 (reference) |  |  |
| Q2 | 0.746 | [0.553,1.005] | 0.054 | 0.824 | [0.604,1.124] | 0.222 |
| Q3 | 0.513 | [0.376,0.701] | 0.000 | 0.600 | [0.433,0.830] | 0.002 |
| Q4 | 0.361 | [0.260,0.502] | 0.000 | 0.500 | [0.354,0.707] | 0.000 |
| P for trend |  |  | 0.000 |  |  | 0.000 |
| minSpO2 |  |  |  |  |  |  |
| Q1 | 1.0 (reference) |  |  | 1.0 (reference) |  |  |
| Q2 | 0.583 | [0.432,0.788] | 0.000 | 0.581 | [0.424,0.795] | 0.001 |
| Q3 | 0.806 | [0.602,1.080] | 0.148 | 0.811 | [0.597,1.102] | 0.181 |
| Q4 | 0.625 | [0.450,0.870] | 0.005 | 0.697 | [0.494,0.984] | 0.040 |
| P for trend | 0.888 | [0.801,0.983] | 0.023 | 0.914 | [0.821,1.018] | 0.101 |
| T901 |  |  |  |  |  |  |
| Q1 | 1.0 (reference) |  |  | 1.00(参考) |  |  |
| Q2 | 0.783 | [0.561,1.092] | 0.150 | 0.700 | [0.495,0.991] | 0.044 |
| Q3 | 1.472 | [1.077,2.011] | 0.015 | 1.335 | [0.964,1.848] | 0.082 |
| Q4 | 1.387 | [1.014,1.898] | 0.041 | 1.165 | [0.838,1.620] | 0.364 |
| P for trend |  |  | 0.002 |  |  | 0.037 |

**Supplementary Table 4.** Stratified analyses for the association between ODI and Mets.

| Subgroup | Predictors:ODI | | | | Outcome:Mets | | |
| --- | --- | --- | --- | --- | --- | --- | --- |
|  |  |  |  |  |  |  |  |
| Gender |  |  |  |  |  |  |  |
| Male | 1025 | 1.045 | [1.029,1.062] | 0.000 | 1.045 | [1.028,1.062] | 0.000 |
| Female | 2415 | 1.061 | [1.044,1.079] | 0.000 | 1.045 | [1.026,1.064] | 0.000 |
| Age (years) |  |  |  |  |  |  |  |
| <40 | 574 | 1.082 | [1.047,1.118] | 0.000 | 1.070 | [1.028,1.113] | 0.001 |
| 40-60 | 1752 | 1.062 | [1.044,1.080] | 0.000 | 1.053 | [1.035,1.072] | 0.000 |
| ≥60 | 1112 | 1.033 | [1.016,1.051] | 0.000 | 1.040 | [1.021,1.058] | 0.000 |
| Alcohol consumption |  |  |  |  |  |  |  |
| Non-drinker | 2876 | 1.062 | [1.049,1.077] | 0.000 | 1.049 | [1.034,1.064] | 0.000 |
| Former drinker | 58 | 1.017 | [0.965,1.072] | 0.526 | 1.061 | [0.917,1.227] | 0.426 |
| Current drinker | 506 | 1.045 | [1.022,1.069] | 0.000 | 1.044 | [1.019,1.069] | 0.000 |
| Smoking |  |  |  |  |  |  |  |
| Non-smoker | 2847 | 1.060 | [1.046,1.074] | 0.000 | 1.049 | [1.034,1.064] | 0.000 |
| Former smoker | 133 | 1.009 | [0.967,1.052] | 0.692 | 1.019 | [0.972,1.069] | 0.432 |
| Current smoker | 460 | 1.056 | [1.030,1.083] | 0.000 | 1.057 | [1.029,1.085] | 0.000 |
| Physical exercise |  |  |  |  |  |  |  |
| 5-7 days per week | 1763 | 1.061 | [1.045,1.077] | 0.000 | 1.052 | [1.034,1.069] | 0.000 |
| 3-4 days per week | 372 | 1.048 | [1.016,1.082] | 0.004 | 1.035 | [0.998,1.073] | 0.063 |
| 1-2 days per week | 394 | 1.057 | [1.022,1.094] | 0.001 | 1.035 | [0.995,1.076] | 0.084 |
| ≤3 days per month | 317 | 1.071 | [1.031,1.112] | 0.000 | 1.059 | [1.012,1.109] | 0.014 |
| Never exercise | 594 | 1.049 | [1.021,1.078] | 0.001 | 1.047 | [1.016,1.078] | 0.003 |

**Supplementary Table 5.** Stratified analyses for the association between MeanspO2 and Mets.

| Subgroup | N | OR | 95%CI | P | OR(adjusted) | 95%CI(adjusted) | P (adjusted) |
| --- | --- | --- | --- | --- | --- | --- | --- |
| Gender |  |  |  |  |  |  |  |
| Male | 1025 | 0.830 | [0.768,0.897] | 0.000 | 0.843 | [0.781,0.911] | 0.000 |
| Female | 2415 | 0.736 | [0.687,0.789] | 0.000 | 0.856 | [0.799,0.918] | 0.000 |
| Age (years) |  |  |  |  |  |  |  |
| <40 | 574 | 0.747 | [0.645,0.866] | 0.000 | 0.824 | [0.719,0.944] | 0.005 |
| 40-60 | 1752 | 0.780 | [0.721,0.845] | 0.000 | 0.815 | [0.753,0.881] | 0.000 |
| ≥60 | 1112 | 0.874 | [0.808,0.945] | 0.001 | 0.867 | [0.800,0.939] | 0.000 |
| Alcohol consumption |  |  |  |  |  |  |  |
| Non-drinker | 2876 | 0.739 | [0.696,0.785] | 0.000 | 0.836 | [0.787,0.888] | 0.000 |
| Former drinker | 58 | 0.918 | [0.756,1.115] | 0.388 | 1.116 | [0.743,1.676] | 0.598 |
| Current drinker | 506 | 0.842 | [0.751,0.944] | 0.003 | 0.852 | [0.758,0.957] | 0.007 |
| Smoking |  |  |  |  |  |  |  |
| Non-smoker | 2847 | 0.743 | [0.699,0.789] | 0.000 | 0.837 | [0.789,0.888] | 0.000 |
| Former smoker | 133 | 0.868 | [0.698,1.079] | 0.203 | 0.843 | [0.660,1.078] | 0.173 |
| Current smoker | 460 | 0.868 | [0.774,0.973] | 0.015 | 0.881 | [0.782,0.993] | 0.038 |
| Physical exercise |  |  |  |  |  |  |  |
| 5-7 days per week | 1763 | 0.803 | [0.752,0.857] | 0.000 | 0.869 | [0.813,0.929] | 0.000 |
| 3-4 days per week | 372 | 0.627 | [0.518,0.759] | 0.000 | 0.697 | [0.568,0.854] | 0.001 |
| 1-2 days per week | 394 | 0.662 | [0.543,0.808] | 0.000 | 0.758 | [0.611,0.941] | 0.012 |
| ≤3 days per month | 317 | 0.720 | [0.588,0.880] | 0.001 | 0.838 | [0.701,1.003] | 0.053 |
| Never exercise | 594 | 0.783 | [0.690,0.889] | 0.000 | 0.860 | [0.746,0.991] | 0.037 |

**Supplementary Table 6.** Stratified analyses for the association between Minspo2 and Mets.

| Subgroup | N | OR | 95%CI | P | OR(adjusted) | 95%CI(adjusted) | P (adjusted) |
| --- | --- | --- | --- | --- | --- | --- | --- |
| Gender |  |  |  |  |  |  |  |
| Male | 1025 | 0.948 | [0.926,0.970] | 0.000 | 0.948 | [0.925,0.971] | 0.000 |
| Female | 2415 | 0.948 | [0.930,0.966] | 0.000 | 0.969 | [0.948,0.989] | 0.003 |
| Age (years) |  |  |  |  |  |  |  |
| <40 | 574 | 0.910 | [0.870,0.952] | 0.000 | 0.933 | [0.887,0.983] | 0.009 |
| 40-60 | 1752 | 0.939 | [0.918,0.959] | 0.000 | 0.950 | [0.929,0.972] | 0.000 |
| ≥60 | 1112 | 0.973 | [0.951,0.996] | 0.021 | 0.968 | [0.945,0.991] | 0.008 |
| Alcohol consumption |  |  |  |  |  |  |  |
| Non-drinker | 2876 | 0.936 | [0.920,0.952] | 0.000 | 0.950 | [0.933,0.968] | 0.000 |
| Former drinker | 58 | 0.988 | [0.913,1.070] | 0.774 | 1.026 | [0.875,1.203] | 0.750 |
| Current drinker | 506 | 0.970 | [0.939,1.002] | 0.069 | 0.969 | [0.936,1.003] | 0.077 |
| Smoking |  |  |  |  |  |  |  |
| Non-smoker | 2847 | 0.945 | [0.929,0.960] | 0.000 | 0.961 | [0.944,0.979] | 0.000 |
| Former smoker | 133 | 1.003 | [0.943,1.066] | 0.927 | 1.001 | [0.932,1.076] | 0.968 |
| Current smoker | 460 | 0.927 | [0.894,0.961] | 0.000 | 0.928 | [0.893,0.964] | 0.000 |
| Physical exercise |  |  |  |  |  |  |  |
| 5-7 days per week | 1763 | 0.948 | [0.929,0.966] | 0.000 | 0.961 | [0.941,0.981] | 0.000 |
| 3-4 days per week | 372 | 0.945 | [0.904,0.988] | 0.013 | 0.957 | [0.910,1.006] | 0.086 |
| 1-2 days per week | 394 | 0.917 | [0.877,0.958] | 0.000 | 0.921 | [0.875,0.969] | 0.002 |
| ≤3 days per month | 317 | 0.912 | [0.867,0.960] | 0.000 | 0.929 | [0.871,0.990] | 0.024 |
| Never exercise | 594 | 0.957 | [0.923,0.993] | 0.018 | 0.967 | [0.928,1.007] | 0.105 |

**Supplementary Table 7.** Stratified analyses for the association between T90 and Mets.

| Subgroup | N | OR | 95%CI | P | OR(adjusted) | 95%CI(adjusted) | P (adjusted) |
| --- | --- | --- | --- | --- | --- | --- | --- |
| Gender |  |  |  |  |  |  |  |
| Male | 1025 | 1.006 | [1.001,1.011] | 0.026 | 1.006 | [1.001,1.011] | 0.021 |
| Female | 2415 | 1.008 | [1.003,1.013] | 0.002 | 1.005 | [1.000,1.010] | 0.045 |
| Age (years) |  |  |  |  |  |  |  |
| <40 | 574 | 1.014 | [1.004,1.023] | 0.004 | 1.009 | [0.999,1.018] | 0.066 |
| 40-60 | 1752 | 1.010 | [1.004,1.017] | 0.001 | 1.009 | [1.003,1.014] | 0.003 |
| ≥60 | 1112 | 1.002 | [0.996,1.007] | 0.524 | 1.002 | [0.997,1.008] | 0.395 |
| Alcohol consumption |  |  |  |  |  |  |  |
| Non-drinker | 2876 | 1.009 | [1.004,1.013] | 0.000 | 1.005 | [1.001,1.010] | 0.016 |
| Former drinker | 58 | 1.007 | [0.993,1.020] | 0.335 | 1.011 | [0.954,1.071] | 0.707 |
| Current drinker | 506 | 1.004 | [0.997,1.012] | 0.265 | 1.005 | [0.997,1.013] | 0.255 |
| Smoking |  |  |  |  |  |  |  |
| Non-smoker | 2847 | 1.009 | [1.004,1.013] | 0.000 | 1.006 | [1.002,1.010] | 0.004 |
| Former smoker | 133 | 0.995 | [0.977,1.013] | 0.589 | 0.998 | [0.978,1.018] | 0.826 |
| Current smoker | 460 | 1.006 | [0.998,1.014] | 0.146 | 1.006 | [0.997,1.015] | 0.170 |
| Physical exercise |  |  |  |  |  |  |  |
| 5-7 days per week | 1763 | 1.006 | [1.001,1.010] | 0.016 | 1.003 | [0.998,1.008] | 0.233 |
| 3-4 days per week | 372 | 1.026 | [1.007,1.047] | 0.009 | 1.021 | [1.001,1.041] | 0.041 |
| 1-2 days per week | 394 | 1.013 | [0.998,1.028] | 0.096 | 1.009 | [0.995,1.024] | 0.213 |
| ≤3 days per month | 317 | 1.008 | [0.999,1.018] | 0.093 | 1.006 | [0.996,1.016] | 0.256 |
| Never exercise | 594 | 1.008 | [0.996,1.019] | 0.192 | 1.007 | [0.995,1.020] | 0.266 |

**Supplementary Table 8.** Mediation analysis for the associations between ODI and Mets

| Independent variable | Mediator | Total effect | | Indirect effect | | Direct effect | | Proportion mediated, % (95% CI) |
| --- | --- | --- | --- | --- | --- | --- | --- | --- |
|  |  | Coefficient (95% CI) | P value | Coefficient (95% CI) | P value | Coefficient (95% CI) | P value |  |
| ODI | SUA/Scr | 0.00689 (0.00530, 0.00849) | <0.001 | 0.00088 (0.00053, 0.00128) | <0.001 | 0.00601 (0.00440, 0.00767) | <0.001 | 12.7 (7.4, 19.9) |
| ODI | INFLA | 0.00697 (0.00541, 0.00851) | <0.001 | 0.00073 (-0.00033, 0.00115) | <0.001 | 0.00625 (0.00473, 0.00781) | <0.001 | 10.3(4.7,17.1) |
| ODI | CRP | 0.00697 (0.00543, 0.00854) | <0.001 | 0.00030 (-0.00009, 0.00058) | 0.004 | 0.00667 (0.00514, 0.00828) | <0.001 | 4.0 (1.2,8.6) |

**Supplementary Table 9.** Association Between Sleep Conditions and MetS Prevalence

| Variable | Crude OR (95% CI) | Adjusted OR (95% CI) | P |
| --- | --- | --- | --- |
| Poor sleep quality (Ref: Normal) | 0.98 (0.78, 1.23) | 0.95 (0.73, 1.22) | 0.6708 |
| PSQI score | 1.00 (0.97, 1.03) | 1.00 (0.96, 1.03) | 0.9088 |
| Sleep quality score | 0.99 (0.85, 1.15) | 1.01 (0.86, 1.20) | 0.8931 |
| Sleep latency score | 1.00 (0.90, 1.10) | 0.95 (0.85, 1.06) | 0.3756 |
| Sleep duration score | 1.04 (0.91, 1.19) | 1.01 (0.88, 1.17) | 0.8576 |
| Sleep efficiency score | 1.04 (0.93, 1.16) | 0.96 (0.85, 1.08) | 0.5005 |
| Sleep disturbance score | 1.16 (0.95, 1.43) | 1.07 (0.85, 1.35) | 0.5547 |
| Use of sleep medication score | 0.42 (0.17, 1.08) | 0.34 (0.12, 0.92) * | 0.033 |
| Daytime dysfunction score | 0.88 (0.78, 0.98) * | 0.95 (0.84, 1.08) | 0.4523 |
| Bedtime (Ref: 20:00~21:00) |  |  |  |
| 21:01~22:00 | 0.82 (0.30, 2.23) | 1.00 (0.33, 3.02) | 0.999 |
| 22:01~23:00 | 0.61 (0.23, 1.58) | 0.89 (0.31, 2.58) | 0.8274 |
| 23:01~0:00 | 0.46 (0.18, 1.19) | 0.78 (0.27, 2.25) | 0.6392 |
| 0:01~1:00 | 0.50 (0.19, 1.31) | 1.01 (0.34, 3.02) | 0.9807 |
| 1:01~4:00 | 0.51 (0.18, 1.43) | 1.14 (0.35, 3.66) | 0.8259 |
| Bedtime trend |  |  | 0.683 |
| Nighttime sleep duration (Ref: 7.1-8 hours) |  |  |  |
| <6 hours |  |  |  |
| 6.1-7 hours | 0.82 (0.30, 2.23) | 1.00 (0.33, 3.02) | 0.7486 |
| 8.1-9 hours | 0.61 (0.23, 1.58) | 0.89 (0.31, 2.58) | 0.2281 |
| 9.1-10 hours | 0.46 (0.18, 1.19) | 0.78 (0.27, 2.25) | 0.3264 |
| Nap duration (Ref: 1-30 minutes) | 0.50 (0.19, 1.31) | 1.01 (0.34, 3.02) | 0.8008 |
| 31-60 minutes |  |  |  |
| 61-90 minutes | 0.89 (0.63, 1.25) | 0.89 (0.63, 1.25) | 0.4984 |
| 91-240 minutes | 1.34 (0.82, 2.20) | 1.34 (0.82, 2.20) | 0.2391 |
| Nap duration trend | 1.12 (0.69, 1.81) | 1.12 (0.69, 1.81) | 0.6531 |
| Poor sleep quality (Ref: Normal) |  |  | 0.367 |

**Supplementary Table 10.** Association Between daytime sleepiness and MetS Prevalence

| Variable | Crude OR (95% CI) | Adjusted OR (95% CI) | P-value |
| --- | --- | --- | --- |
| Sitting and reading | 1.10 (0.94, 1.29) | 1.06 (0.89, 1.26) | 0.518 |
| Watching TV | 1.13 (0.97, 1.33) | 1.07 (0.90, 1.27) | 0.475 |
| Sitting quietly in a public place | 1.10 (0.90, 1.35) | 1.08 (0.86, 1.35) | 0.498 |
| Sitting in a car for an hour | 0.95 (0.84, 1.08) | 1.02 (0.89, 1.16) | 0.823 |
| Lying down to rest in the afternoon | 1.08 (0.94, 1.23) | 1.09 (0.94, 1.26) | 0.261 |
| Sitting and talking to someone | 0.82 (0.55, 1.23) | 0.74 (0.49, 1.14) | 0.176 |
| Sitting quietly after lunch (without alcohol) | 0.98 (0.85, 1.13) | 0.99 (0.85, 1.15) | 0.845 |
| Sitting in a car, stopped for a few minutes in traffic | 1.01 (0.82, 1.26) | 1.06 (0.84, 1.34) | 0.602 |
| ESS score (continuous variable) | 1.01 (0.98, 1.05) | 1.01 (0.98, 1.05) | 0.467 |
| ESS (categorical variable) |  |  |  |
| <9 (reference) | 1.0 | 1.0 |  |
| ≥9 | 1.01 (0.53, 1.91) | 0.92 (0.46, 1.84) | 0.819 |

**Supplementary Table 11.**Association Between insomnia and MetS Prevalence

| Variable | Crude OR (95% CI) | Adjusted OR (95% CI) | P-value |
| --- | --- | --- | --- |
| Difficulty falling asleep | 1.01 (0.94, 1.08) | 0.98 (0.90, 1.05) | 0.528 |
| Difficulty maintaining sleep | 1.01 (0.94, 1.09) | 0.97 (0.90, 1.06) | 0.526 |
| Early awakening | 1.04 (0.96, 1.12) | 1.03 (0.94, 1.12) | 0.513 |
| Satisfaction with current sleep pattern | 0.94 (0.82, 1.07) | 0.97 (0.84, 1.12) | 0.659 |
| Impact of sleep problems on daily life functioning | 0.89 (0.76, 1.04) | 0.92 (0.78, 1.09) | 0.318 |
| Extent to which insomnia problems affect or impair quality of life compared to others | 0.87 (0.76, 1.00) | 0.91 (0.79, 1.06) | 0.243 |
| Concern/distress about current sleep problems | 1.00 (0.89, 1.13) | 1.05 (0.92, 1.20) | 0.448 |
| ISI score (continuous variable) | 1.00 (0.98, 1.02) | 1.00 (0.98, 1.02) | 0.718 |
| ISI score (categorical variable) |  |  |  |
| ≤7 (reference) | 1.0 | 1.0 |  |
| >7 | 1.02 (0.81, 1.30) | 0.99 (0.77, 1.29) | 0.954 |

**Supplementary Table 12.**Association Between SDB-Related Symptoms and MetS Prevalence

| Variable | Crude OR (95% CI) | Adjusted OR (95% CI) | P-value |
| --- | --- | --- | --- |
| Chest discomfort |  |  |  |
| No | 1.0 | 1.0 |  |
| Occasionally | 1.00 (0.79, 1.27) | 0.89 (0.69, 1.15) | 0.389 |
| Frequently | 1.17 (0.75, 1.83) | 0.93 (0.58, 1.51) | 0.780 |
| Dizziness and headache |  |  |  |
| No | 1.0 | 1.0 |  |
| Occasionally | 1.08 (0.85, 1.37) | 1.10 (0.85, 1.43) | 0.463 |
| Frequently | 0.87 (0.60, 1.25) | 0.87 (0.58, 1.29) | 0.478 |
| Dry mouth upon waking |  |  |  |
| No | 1.0 | 1.0 |  |
| Occasionally | 1.13 (0.86, 1.49) | 1.19 (0.89, 1.60) | 0.240 |
| Frequently | 1.22 (0.93, 1.61) | 1.20 (0.89, 1.62) | 0.232 |
| Loud snoring |  |  |  |
| No | 1.0 | 1.0 |  |
| Yes | 2.05 (1.63, 2.58) | 2.02 (1.57, 2.59) | <0.0001 |
| Unrefreshing sleep |  |  |  |
| No | 1.0 | 1.0 |  |
| Yes | 0.76 (0.60, 0.97) | 0.88 (0.68, 1.14) | 0.319 |
| Observed apnea |  |  |  |
| No | 1.0 | 1.0 |  |
| Yes | 1.69 (1.18, 2.43) | 1.62 (1.09, 2.40) | 0.017 |

**Supplementary Table 13.**Clinicodemographic participant characteristics in the total sample and each cluster

| Variable | Cluster 1 | Cluster 2 | Cluster 3 | Cluster 4 | P-value |
| --- | --- | --- | --- | --- | --- |
| N | 347 | 625 | 251 | 260 |  |
| Age, years, mean (SD) | 60.6 ± 10.8 | 55.1 ± 12.4 | 55.3 ± 13.9 | 51.3 ± 13.6 | <0.001 |
| BMI, kg/m2, mean (SD) | 24.5 ± 3.3 | 24.6 ± 3.2 | 24.0 ± 3.7 | 24.8 ± 3.7 | 0.051 |
| Neck circumference, cm, mean (SD) | 34.7 ± 3.4 | 35.4 ± 3.5 | 34.3 ± 3.4 | 34.9 ± 4.0 | <0.001 |
| Waist circumference, cm, mean (SD) | 85.7 ± 9.0 | 85.6 ± 9.9 | 83.5 ± 10.0 | 85.0 ± 11.6 | 0.035 |
| ODI, events/h,mean (SD) | 11.3 ± 7.2 | 11.4 ± 6.9 | 11.4 ± 8.0 | 11.8 ± 7.9 | 0.876 |
| Average SpO2, %,mean (SD) | 95.6 ± 2.1 | 95.8 ± 1.6 | 95.5 ± 2.3 | 95.9 ± 1.8 | 0.074 |
| T90,%,mean (SD) | 13.5 ± 30.7 | 12.7 ± 26.5 | 16.6 ± 39.8 | 12.2 ± 22.2 | 0.287 |
| Sex |  |  |  |  | <0.001 |
| Male | 115 (33.1%) | 294 (47.0%) | 78 (31.1%) | 101 (38.8%) |  |
| Female | 232 (66.9%) | 331 (53.0%) | 173 (68.9%) | 159 (61.2%) |  |


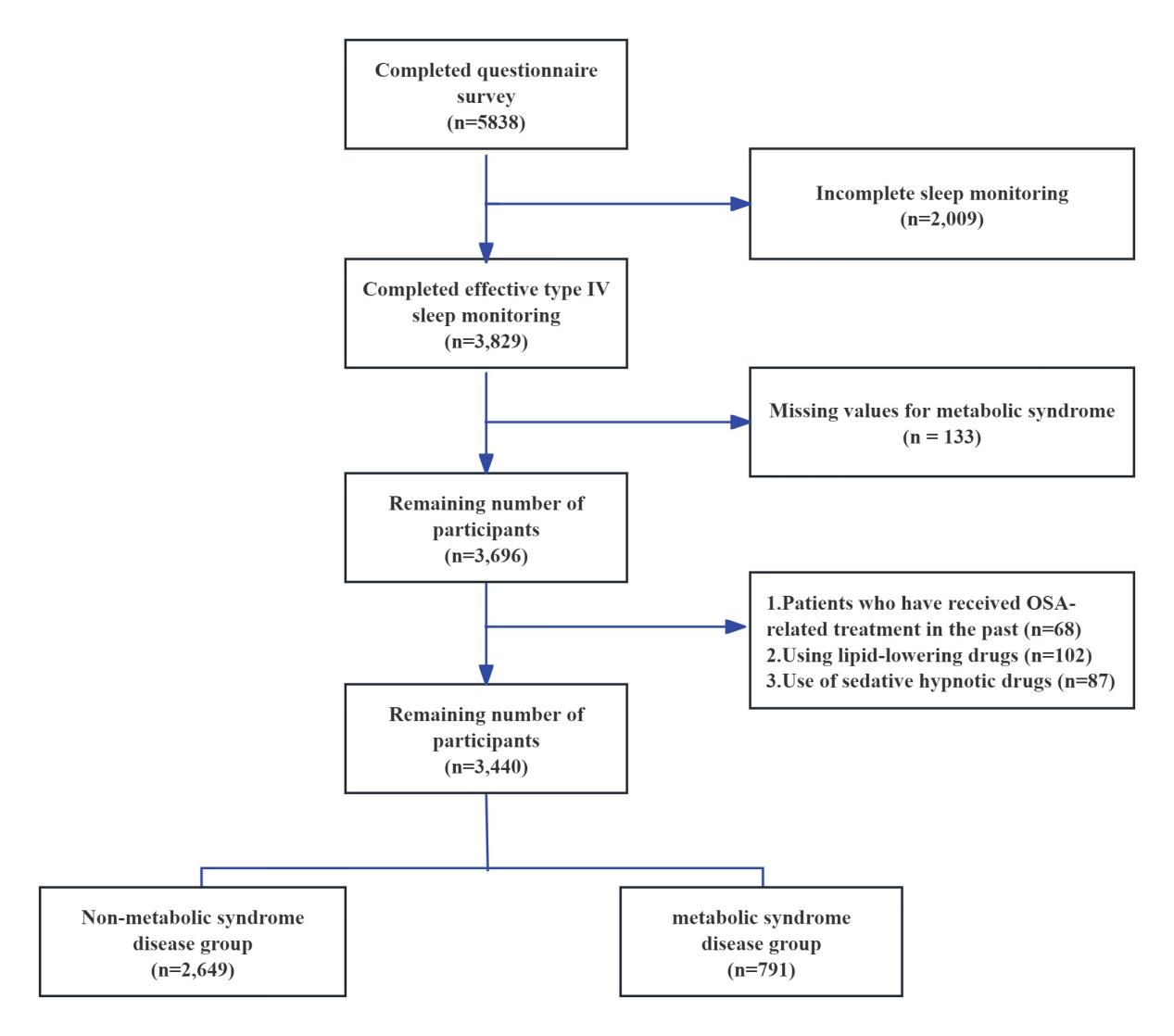


**Figure S1.**  Flowchart of participant selection

**Figure S2.** Type IV intelligent wearable sleep monitoring devices and method of use

(A)

**
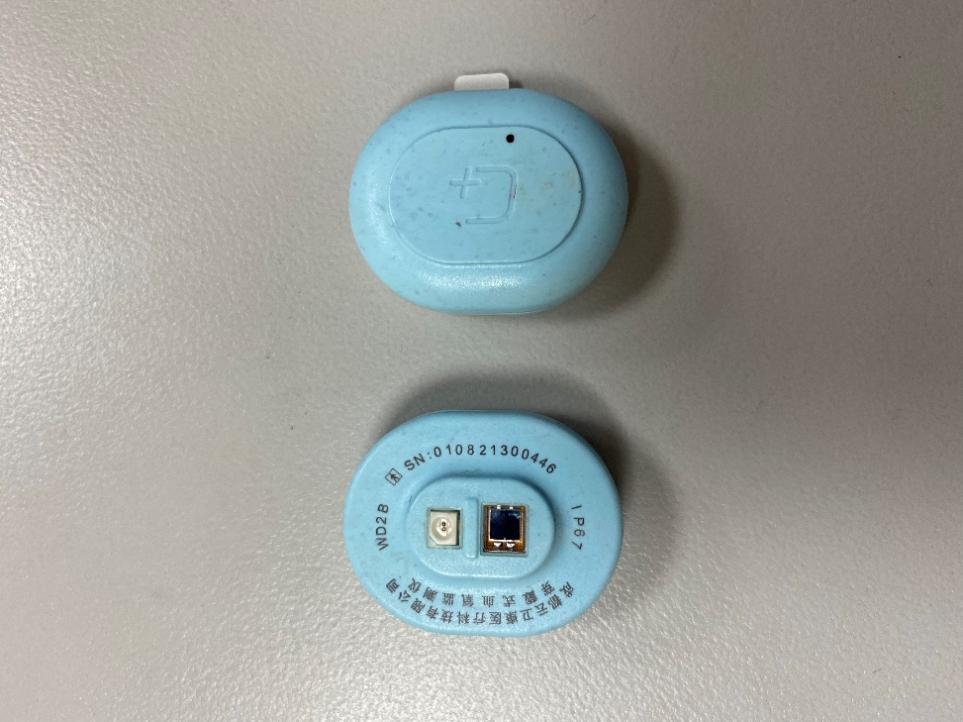
**

(B)

**
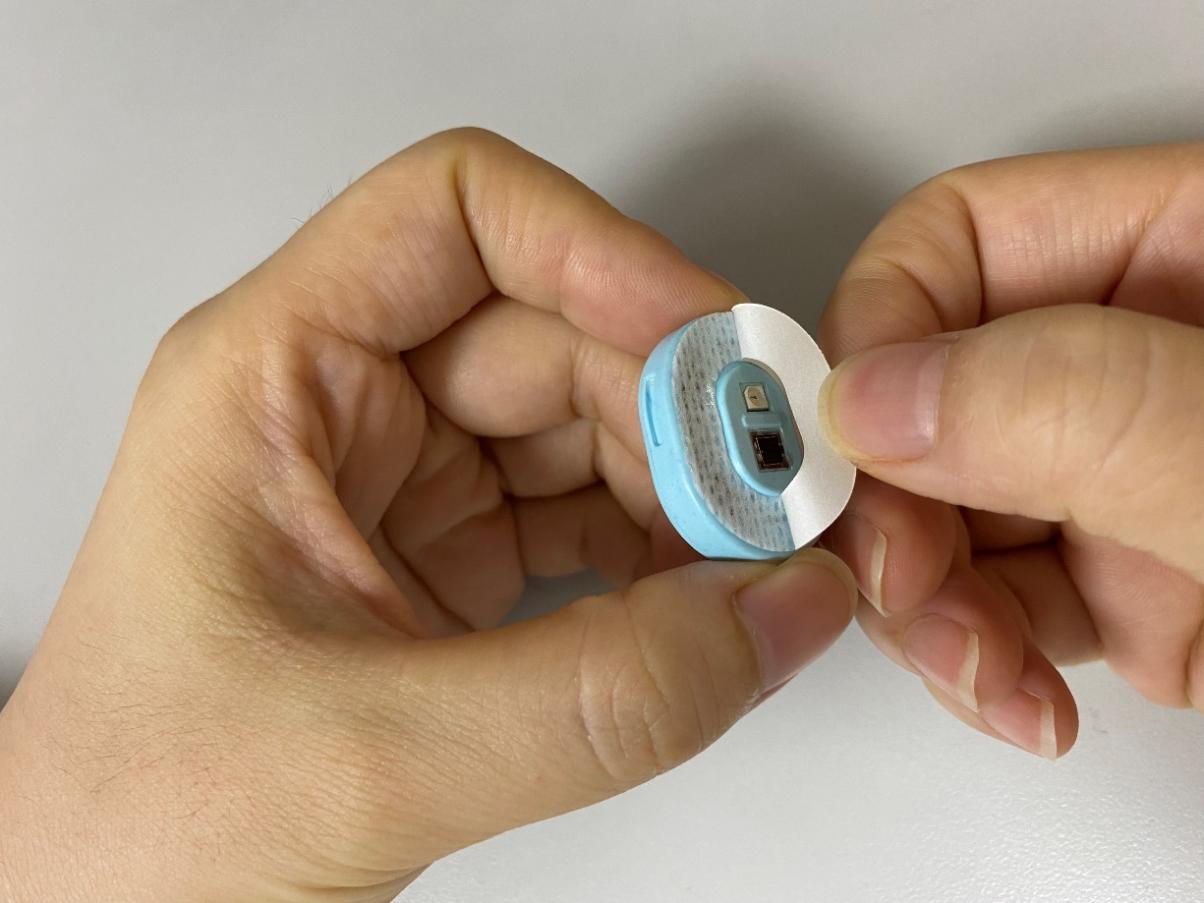
**

(C)

**
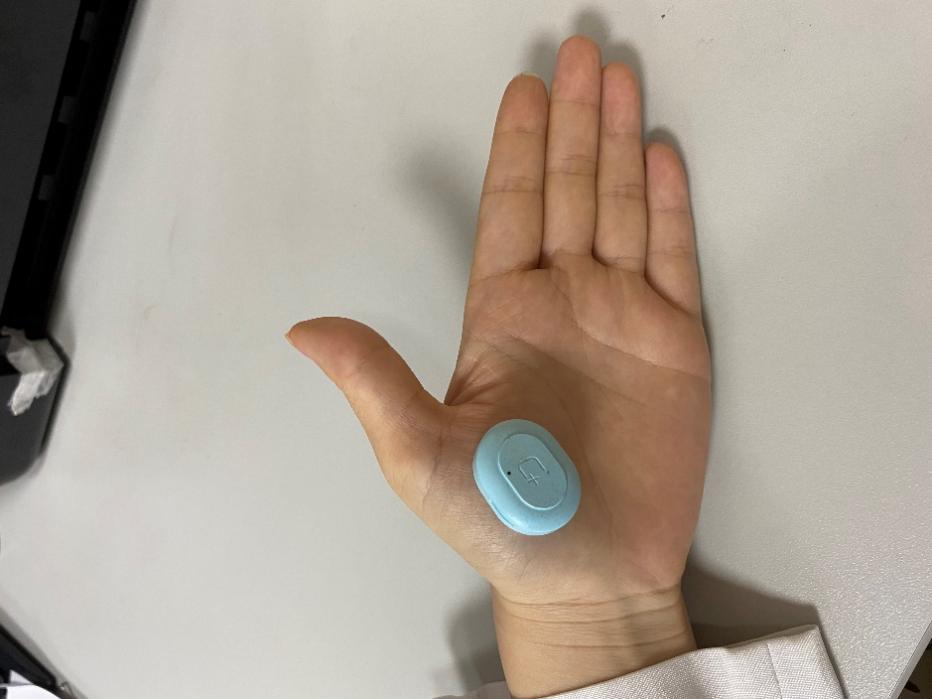
**

(A) Front and back photos of intelligent wearable sleep monitoring devices

(B) Double-sided adhesive is attached to the side with the photoelectric reflex sensor

(C) The device is tightly adhered to the position of palmar thenar major muscles with double-sided adhesive

**Figure S3.** Validation of intelligent wearable sleep monitoring devices by comparison against polysomnography

(B)

(A)


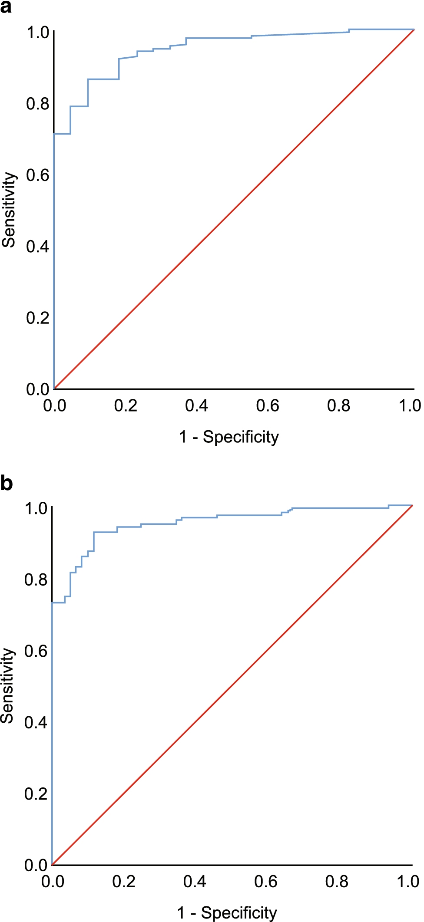

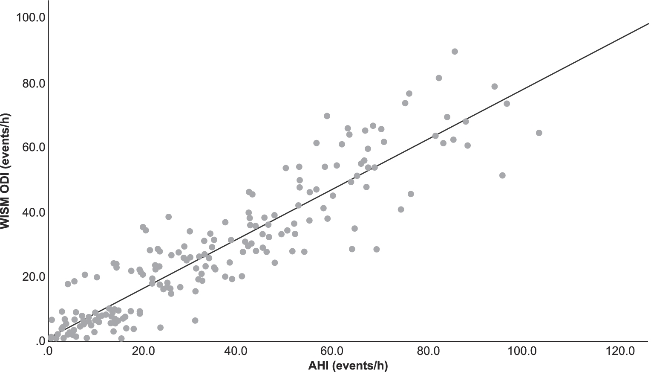


**(A) ROC curve of SDB diagnosed by WISM at AHI threshold ≥ 5 times/hour as defined by PSG. (B) AHI and WISM ODI conformance analysis.**

Note: Figures (A) and (B) are from the previously published study of our research team; Adapted with permission from Xu Y, Ou Q, Cheng Y, et al. Comparative study of a wearable intelligent sleep monitor and polysomnography monitor for the diagnosis of obstructive sleep apnea. *Sleep Breath*, 2022.^1^

Our research team validated the wearable intelligent sleep monitoring device (WISM) by comparison against polysomnography (PSG) at the Sleep Center of Guangdong Provincial People's Hospital from July 2020 to March 2021. In total, 196 participants completed both PSG and WISM monitoring at the same time. The ODI had a strong correlation with the AHI from the PSG (R ^2^ =0.843, P < 0.001, Figure (B)).

SDB= sleep-disordered breathing; ROC= receiver-operating characteristic; WISM= wearable intelligent sleep monitor; AHI apnea–hypopnea index; ODI= oxygen desaturation index; PSG= polysomnography

References

[1] Xu Y, Ou Q, Cheng Y, et al. Comparative study of a wearable intelligent sleep monitor and polysomnography monitor for the diagnosis of obstructive sleep apnea [J]. Sleep Breath, 2022. DOI: 10.1007/s11325-022-02599-x.
